# Insulin resistance assessed by estimated glucose disposal rate and risk of incident cardiovascular diseases among individuals without diabetes: findings from a nationwide, population based, prospective cohort study

**DOI:** 10.1186/s12933-024-02256-5

**Published:** 2024-06-06

**Authors:** Zenglei Zhang, Lin Zhao, Yiting Lu, Yan Xiao, Xianliang Zhou

**Affiliations:** Department of Cardiology, National Center for Cardiovascular Diseases, Fuwai Hospital, Chinese Academy of Medical Sciences and Peking Union Medical College, No.167, Beilishi Road, 100037 Xicheng DistrictBeijing, China

**Keywords:** Insulin resistance, Cardiovascular diseases, Estimated glucose disposal rate, Non-diabetes, Predictive performance

## Abstract

**Background:**

Recent studies have suggested that insulin resistance (IR) contributes to the development of cardiovascular diseases (CVD), and the estimated glucose disposal rate (eGDR) is considered to be a reliable surrogate marker of IR. However, most existing evidence stems from studies involving diabetic patients, potentially overstating the effects of eGDR on CVD. Therefore, the primary objective of this study is to examine the relationship of eGDR with incidence of CVD in non-diabetic participants.

**Method:**

The current analysis included individuals from the China Health and Retirement Longitudinal Study (CHARLS) who were free of CVD and diabetes mellitus but had complete data on eGDR at baseline. The formula for calculating eGDR was as follows: eGDR (mg/kg/min) = 21.158 − (0.09 × WC) − (3.407 × hypertension) − (0.551 × HbA1c) [WC (cm), hypertension (yes = 1/no = 0), and HbA1c (%)]. The individuals were categorized into four subgroups according to the quartiles (Q) of eGDR. Crude incidence rate and hazard ratios (HRs) with 95% confidence intervals (CIs) were computed to investigate the association between eGDR and incident CVD, with the lowest quartile of eGDR (indicating the highest grade of insulin resistance) serving as the reference. Additionally, the multivariate adjusted restricted cubic spine (RCS) was employed to examine the dose–response relationship.

**Results:**

We included 5512 participants in this study, with a mean age of 58.2 ± 8.8 years, and 54.1% were female. Over a median follow-up duration of 79.4 months, 1213 incident CVD cases, including 927 heart disease and 391 stroke, were recorded. The RCS curves demonstrated a significant and linear relationship between eGDR and all outcomes (all *P* for non-linearity > 0.05). After multivariate adjustment, the lower eGDR levels were founded to be significantly associated with a higher risk of CVD. Compared with participants with Q1 of eGDR, the HRs (95% CIs) for those with Q2 − 4 were 0.88 (0.76 − 1.02), 0.69 (0.58 − 0.82), and 0.66 (0.56 − 0.79). When assessed as a continuous variable, per 1.0-SD increase in eGDR was associated a 17% (HR: 0.83, 95% CI: 0.78 − 0.89) lower risk of CVD, with the subgroup analyses indicating that smoking status modified the association (*P* for interaction = 0.012). Moreover, the mediation analysis revealed that obesity partly mediated the association. Additionally, incorporating eGDR into the basic model considerably improve the predictive ability for CVD.

**Conclusion:**

A lower level of eGDR was found to be associated with increased risk of incident CVD among non-diabetic participants. This suggests that eGDR may serve as a promising and preferable predictor and intervention target for CVD.

**Supplementary Information:**

The online version contains supplementary material available at 10.1186/s12933-024-02256-5.

## Introduction

Cardiovascular diseases (CVD), have emerged as the leading cause of morbidity and premature death worldwide [1, 2], particularly in developing countries. According to estimates from the Global Burden of Disease Study 2017, approximately 17.8 million premature death and 330.2 million years of life can be attribute to CVD globally [3], imposing a substantial economic burden on healthcare systems and presenting an urgent challenge for public health. Despite significant advancements in the prevention, diagnosis, and treatment of CVD in recent years, the global incidence of CVD continues to rise [1]. In clinical practice, it is increasingly common to encounter individuals experiencing new-onset CVD despite well-controlled traditional cardiovascular risk factors, suggesting the presence of residual CVD risks.

Insulin resistance (IR), a pathophysiological condition characterized by decreased responsiveness of target organs or tissues to insulin [4], leads to impairments in utilizing blood glucose [5], is widely recognized as a significant contributor to CVD and mortality [6, 7]. Although the precise biological mechanisms linking IR to CVD remain unclear, the possible explanations have been proposed, such as metabolic disturbance, oxidative stress, endothelial impairment, exaggerated inflammation, and inappropriately activated renin–angiotensin–aldosterone system (RAAS) [8–10]. Given these adverse effects, several methods have been developed to evaluated IR. While the hyperinsulinemic-euglycemic (HIEG) clamp is considered the gold standard for identifying IR, its clinical utility and feasibility in large-scale epidemiological investigations are limited due to its time-consuming and burdensome nature [11]. Similarly, the homeostasis model assessment for insulin resistance (HOMA-IR) is not suitable for large population-based cohort studies due to its cost and complexity [12]. Recently, a simpler indicator, eGDR, have emerged as reliable surrogate markers of IR. Previous studies have shown that lower eGDR is associated with a higher risk of stroke, coronary artery disease, and all-cause mortality [6, 7, 13–15]. However, these studies primarily focus on diabetic individuals, potentially exaggerating or confounding the role of IR, and often involve limited sample sizes. While a recent study suggests a correlation between eGDR and increased cardiovascular disease risk in the general population, and this relationship is not modified by diabetic status [16], prior research consistently indicates significant heterogeneity between diabetic and non-diabetic populations. Diabetics exhibit more comorbidities, higher cardiovascular risk, and mortality [17–19]. Ren et al. also indicates that the non-diabetic group is more sensitive to eGDR [16]. Moreover, the association between eGDR and incident CVD has not been thoroughly evaluated.

Therefore, to address these knowledge gaps, we enrolled participants from CHARLS, a nationwide, population based, prospective cohort study, to explore the relationship between eGDR and incidence of CVD among individuals without diabetes. Additionally, considering the bidirectional relationship between IR and obesity [20], we explored whether obesity mediated the association of eGDR and CVD. Furthermore, we assessed whether incorporating eGDR into the basic model could enhance its predictive power.

## Methods

### Study design and population

We extracted data from the CHARLS cohort study of Chinese residents aged 45 years and older [21]. Detailed information regarding the study design and enrolled criteria have been previously reported [21]. In brief, the study conducted baseline survey from June 2011 to March 2012, and a total of 17,708 individuals residing in 10,257 households were selected as nationally representative samples. These participants underwent regular follow-ups every two years through face-to-face interviews conducted by trained interviewers using computer-assisted guidance. Four subsequent follow-up waves were conducted in 2013, 2015, 2018, and 2020, but data from the latest survey wave have not yet been released.

In this study, a total of 5512 participants were included in the analysis, and further categorized into four subgroups based on the quartiles (Q) of eGDR. The other 12,196 participants were excluded from the analysis for the following reasons: no available data on eGDR (n = 7770); diagnosed with CVD at baseline (n = 1397); diagnosed with diabetes in 2011 (n = 1424); diagnosed with cancer at baseline (n = 75); aged < 45 years old, or unavailable data on age (n = 298); missing information on CVD at baseline or lost to follow-up (n = 1232) (Supplementary file 1, Figure S1).

### Data collection and definition

The CHARLS investigators collected variables according to pre-specified standards. The participants’ blood pressure (BP) was calculated as the average of the three-time BP measurements taken in a sitting position after resting for five minutes. Body mass, height and waist circumference (WC) also were measured while participants wore lightweight clothes and no shoes. Blood samples were collected from CHARLS participants at baseline after an overnight fast by professional staff, stored at − 20 °C, and transported to Beijing, where further measurements were conducted following standard procedures. Biochemical parameters included high-sensitivity C-reactive protein (hsCRP), blood urea nitrogen, serum creatinine, glycosylated hemoglobin A1c (HbA1c), and lipid profiles.

Hypertension was defined as follows: a self-reported hypertension based on physician diagnosis, and/or any use of antihypertensive drugs, and/or BP ≥ 140/90 mmHg [22]. Diabetes was defined based on a self-reported physician diagnosis, use of hypoglycemic drugs, or FBG ≥ 126 mg/dL, and/or an HbA1c level ≥ 6.5% at baseline [23]. Kidney disease was defined as self-reported physician diagnosis and estimated glomerular filtration rate < 60 ml/minute/1.73 m^2^, following the methodology used in the previous CHARLS study [24]. The body mass index (BMI) was calculated as: BMI(kg/m^2^) = body mass/height^2^. The Qinling Mountains-Huaihe River Line was used to delineate the North and South areas [25]. Obesity was defined as BMI ≥ 28 kg/m^2^.

### Ascertainment of exposure and outcomes

The exposure of this study was eGDR at baseline. The formula for calculating eGDR was as follows: eGDR (mg/kg/min) = 21.158 − (0.09 × WC) − (3.407 × hypertension) − (0.551 × HbA1c) [WC (cm), hypertension (yes = 1/no = 0), and HbA1c (%)].

The primary outcome of interest was CVD, including heart disease and stroke. Consistent with established precedents [26, 27], incident CVD was determined based on self-reports where participants confirmed having received a definite diagnosis of CVD from physicians. Participants were followed from baseline (2011) until the occurrence of stroke or cardiac events or the most recent survey (2018), whichever occurred first.

### Statistical analysis

RStudio 4.2.1 software was employed for all statistical analyses. A two-tailed *P* value < 0.05 was considered statistically significant. Continuous variables were presented as mean ± standard deviation (SD) or median (interquartile range) where appropriate. Baseline data comparisons for normally and skewed distributed data were performed using analysis of variance and Kruskal–Wallis H test, respectively. Categorical variables were expressed as counts and percentages, with differences determined through chi‐square tests. We conducted trend tests using the median value of each quartile of eGDR. The multiple imputation method was used to impute the missing values.

Kaplan–Meier curves were generated to illustrate the cumulative incidence of CVD, with differences compared using the log-rank test. Incidence rates of CVD events were reported as per 1000 person-years. The three Cox proportional hazards models were fitted to estimate hazard ratios (HRs) between eGDR and CVD, along with the corresponding 95% confidence intervals (CIs). The proportional hazards assumption of each included variates in the models was checked with the Schoenfeld residual test, and no violations were observed. Model 1 was an unadjusted model; Model 2 adjusted for age, sex, rural residence, marital status, education, smoking, and alcohol consumption status; Model 3 further adjusted region, total cholesterol (TC), high density lipoprotein (HDL), triglyceride (TG), low density lipoprotein (LDL), BUN, uric acid (UA), hsCRP, hemoglobin, chronic kidney disease, and obesity. To investigate the dose–response relationship between eGDR and the incidence of CVD, RCS based on Cox regression models was employed, adjusting covariates in model 3, and the eGDR value at HR = 1 was treated as the reference. The receiver operating characteristics curves were established to assess the predictive value of eGDR on incidence of CVD, and the *C*-statistic, was used to quantify [4, 28]. To further estimate additional the predictive power beyond the basic models, the net reclassification improvement (NRI) and integrated discrimination improvement (IDI) index were computed [29].

Subgroup analyses were conducted to assess the effects of eGDR (both continuous and categorical) on the incidence of CVD in several subgroups, including age (< / ≥ 60 years), sex (male/female), smoking (yes/no), and drinking (yes/no). Several sensitivity analyses were conducted to evaluate the robustness of main findings. First, the analysis was repeated among participants with normal glucose status. Second, eGDR was redefined based on hypertension (130/80 mm Hg). Third, participants who developed CVD during or before Survey 2 were excluded to reduce the potential reverse causation bias. Finally, the association of eGDR with CVD was examined among non-DM participants (defined DM based on FBG and HbA1c).

## Results

### Participants characteristics

The comparison of baseline characteristics stratified by quartiles of eGDR (Q1:6.58 ± 0.69; Q2: 9.02 ± 0.89; Q3: 10.76 ± 0.27; Q4: 11.73 ± 0.40) is presented in Table 1. A total of 5512 subjects (mean age: 58.16 ± 8.82 years) with 54.1% female were included in this study. The mean age, proportion of female, systolic BP, diastolic BP, BMI, WC, levels of hemoglobin, HbA1c, TC, TG, LDL, UA, and hsCRP, proportion of obesity all decreased with increasing eGDR (all *P* < 0.001). However, individuals with higher levels of eGDR tended to live in rural and south areas, and higher proportion of current smoking. The proportion of alcohol consumption was the highest in the Q4 of eGDR (42.7%). The baseline characteristics of included individuals according to CVD were presented in Supplementary file 1, Table S1.

**Table 1:** Baseline characteristics of participants stratified by quartiles of estimated glucose disposal rate

Characteristics	Overall	Quartiles of eGDR				
		Quartile 1	Quartile 2	Quartile 3	Quartile 4	*P* value
n	5512	1370	1371	1369	1402	
eGDR	9.52 ± 2.05	6.58 ± 0.69	9.02 ± 0.89	10.76 ± 0.27	11.73 ± 0.40	< 0.001
Age, years	58.16 ± 8.82	59.82 ± 9.05	58.81 ± 9.20	56.66 ± 8.09	57.39 ± 8.57	< 0.001
Female, n (%)	2983 (54.1)	788 (57.5)	750 (54.7)	717 (52.4)	728 (51.9)	0.012
SBP^b^, mmHg	127.67 ± 20.60	146.90 ± 19.73	131.70 ± 20.22	117.41 ± 11.15	115.42 ± 12.05	< 0.001
DBP^b^, mmHg	74.65 ± 12.05	83.99 ± 11.94	76.83 ± 11.39	69.97 ± 8.71	68.19 ± 8.93	< 0.001
BMI^b^, kg/m^2^	23.21 ± 3.48	25.61 ± 3.30	23.98 ± 3.72	22.94 ± 2.20	20.39 ± 2.13	< 0.001
WC, cm	84.47 ± 9.73	92.50 ± 7.50	86.94 ± 10.72	84.22 ± 3.57	74.44 ± 4.75	< 0.001
Rural residence, n (%)	3764 (68.3)	869 (63.4)	906 (66.1)	950 (69.4)	1039 (74.1)	< 0.001
Region^a^, n (%)						< 0.001
North	2381 (43.2)	706 (51.5)	602 (43.9)	596 (43.5)	477 (34.0)	
South	3131 (56.8)	664 (48.5)	769 (56.1)	773 (56.5)	925 (66.0)	
Education, n (%)						0.634
Junior high school and below	5003 (90.8)	1259 (91.9)	1246 (90.9)	1229 (89.8)	1269 (90.5)	
Senior high school	470 (8.5)	103 (7.5)	116 (8.5)	130 (9.5)	121 (8.6)	
Tertiary	39 (0.7)	8 (0.6)	9 (0.7)	10 (0.7)	12 (0.9)	
Marital status, n (%)						0.102
Married and living with spouse	4708 (85.4)	1156 (84.4)	1165 (85.0)	1197 (87.4)	1190 (84.9)	
Others	804 (14.6)	214 (15.6)	206 (15.0)	172 (12.6)	212 (15.1)	
Alcohol consumption, n (%)	2272 (41.2)	556 (40.6)	564 (41.1)	554 (40.5)	598 (42.7)	0.628
Smoking, n (%)	2128 (38.6)	482 (35.2)	535 (39.0)	531 (38.8)	580 (41.4)	0.010
Hemoglobin^b^, g/dL	14.34 ± 2.20	14.70 ± 2.31	14.46 ± 2.26	14.22 ± 2.10	13.99 ± 2.07	< 0.001
FBG, mg/dL	99.98 ± 11.63	101.91 ± 11.53	100.91 ± 11.50	99.45 ± 11.70	97.68 ± 11.35	0.916
HbA1c, %	5.10 ± 0.40	5.18 ± 0.40	5.13 ± 0.40	5.12 ± 0.37	4.98 ± 0.38	< 0.001
TC, mg/dL	192.37 ± 36.96	198.83 ± 37.55	193.50 ± 36.22	191.95 ± 38.05	185.36 ± 34.75	< 0.001
TG, mg/dl	100.89 (72.57 − 144.26)	120.36 (84.07 − 169.92)	105.31 (75.22 − 152.22)	97.35 (71.68 − 135.40)	84.96 (62.83 − 116.82)	< 0.001
HDL, mg/dL	52.50 ± 14.97	48.69 ± 13.68	51.26 ± 15.08	53.08 ± 14.59	56.86 ± 15.28	< 0.001
LDL^b^, mg/dL	116.88 ± 33.53	122.44 ± 34.28	116.91 ± 33.66	117.49 ± 33.70	110.83 ± 31.45	< 0.001
BUN, mg/dL	15.64 ± 4.38	15.64 ± 4.34	15.58 ± 4.50	15.66 ± 4.42	15.69 ± 4.27	< 0.001
UA, mg/dL	4.38 ± 1.20	4.63 ± 1.22	4.48 ± 1.25	4.26 ± 1.15	4.16 ± 1.11	< 0.001
hsCRP, mg/L	0.94 (0.52 − 1.96)	1.31 (0.71 − 2.56)	0.99 (0.55 − 2.02)	0.84 (0.49 − 1.75)	0.72 (0.41 − 1.41)	< 0.001
Serum creatinine, mg/dL	0.77 ± 0.18	0.78 ± 0.19	0.77 ± 0.19	0.76 ± 0.18	0.76 ± 0.16	0.002
Kidney disease, n (%)	368 (6.7)	103 (7.6)	94 (6.9)	84 (6.2)	87 (6.3)	0.436
Obesity, n (%)	556 (10.1)	311 (22.7)	200 (14.6)	31 (2.3)	14 (1.0)	< 0.001

*BMI* body mass index, *BUN* blood urea nitrogen, *DBP* diastolic blood pressure, *DM* diabetes mellitus, *eGDR* estimated glucose disposal rate, *FBG* fasting blood glucose, *HbA1c* glycosylated hemoglobin A1c, *HDL* high density lipoprotein, *hsCRP* high-sensitivity C-reactive protein, *LDL* low density lipoprotein, *SBP* systolic blood pressure, *TC* total cholesterol, *TG* triglycerides, *UA* uric acid, *WC* waist circumference

^a^Region was divided into north and south based on the Qinling Mountains-Huaihe River Line

^b^Missing data: 43 for systolic blood pressure, 44 for diastolic blood pressure, 1 for LDL, 91 for hemoglobin, 49 for BMI

### Associations of baseline eGDR with incident CVD

**Fig. 1:**
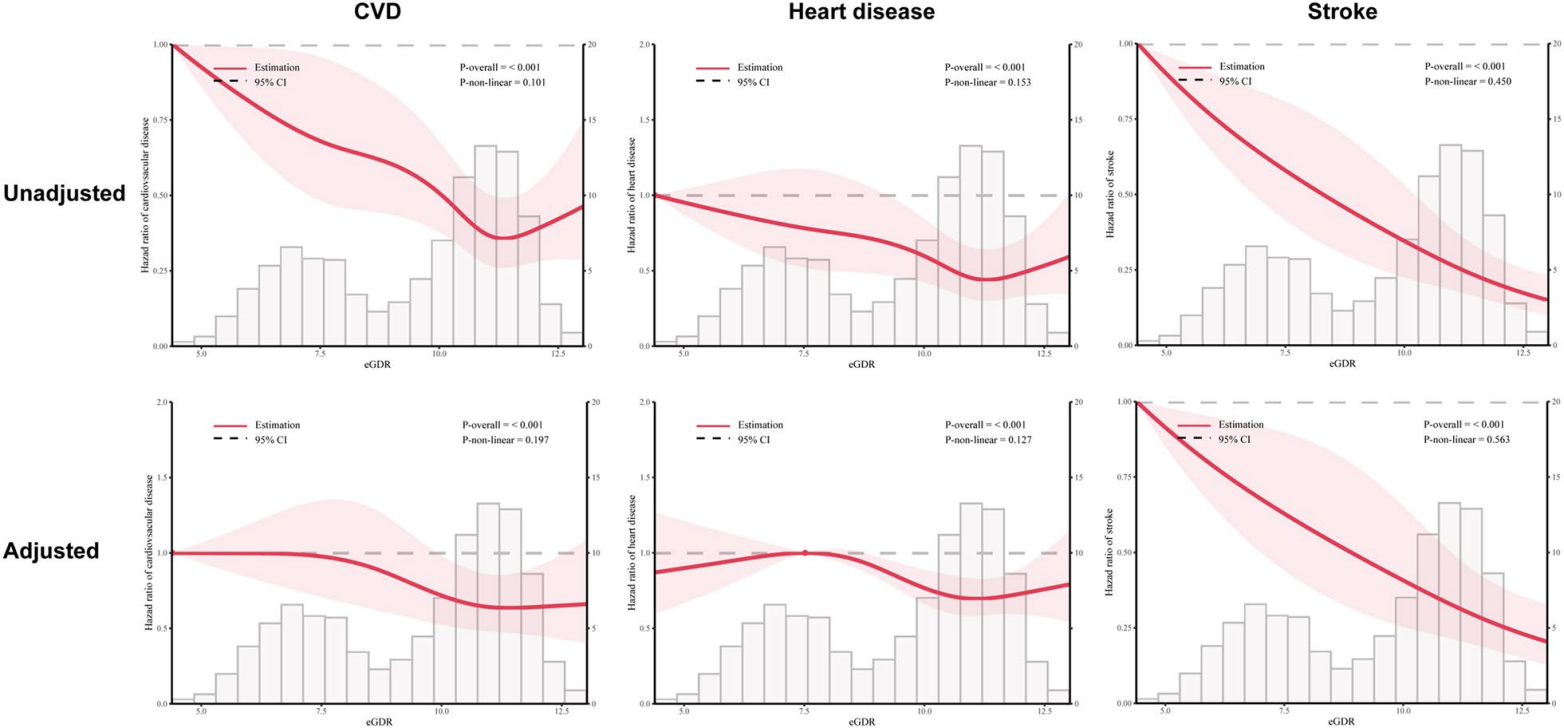
Restricted cubic spline curves for CVD according to the eGDR. Hazard ratios are indicated by solid lines and 95% CIs by shaded areas. The horizontal dotted line represents the hazard ratio of 1.0. The adjusted models adjusted age, sex, rural residence, marital status, education, smoking, alcohol consumption status, region, TC, HDL, TG, LDL, BUN, UA, hsCRP, hemoglobin, chronic kidney disease, and obesity

During a median follow-up period of 79.4 months, there were 1213 (22.0%) cases of incident CVD, including 927 (16.8%) cases of heart disease and 391 (7.1%) case of stroke, were recorded. The incidences of CVD among participants with Q1 − 4 were 46.3, 36.6, 26.8, and 24.1 per 1000 person-years, respectively. The dose–response curves between eGDR and CVD were presented in Fig. 1. These RCS curves suggested a significant and linear relationship between eGDR and the incidence of CVD, heart disease, and stroke in with or without adjusting for covariates (all *P* for overall < 0.001 and *P* for non-linear > 0.05). After fully adjusted covariates (Model 3), per 1.0-SD increasement in eGDR was associated with a 17% (HR: 0.83, 95% CI: 0.78 − 0.89) lower risk for CVD, a 13% (HR: 0.87, 95% CI: 0.81 − 0.94) decreased risk for heart disease, and 30% (HR: 0.63 − 0.78) lower risk for stroke.

**Fig. 2:**
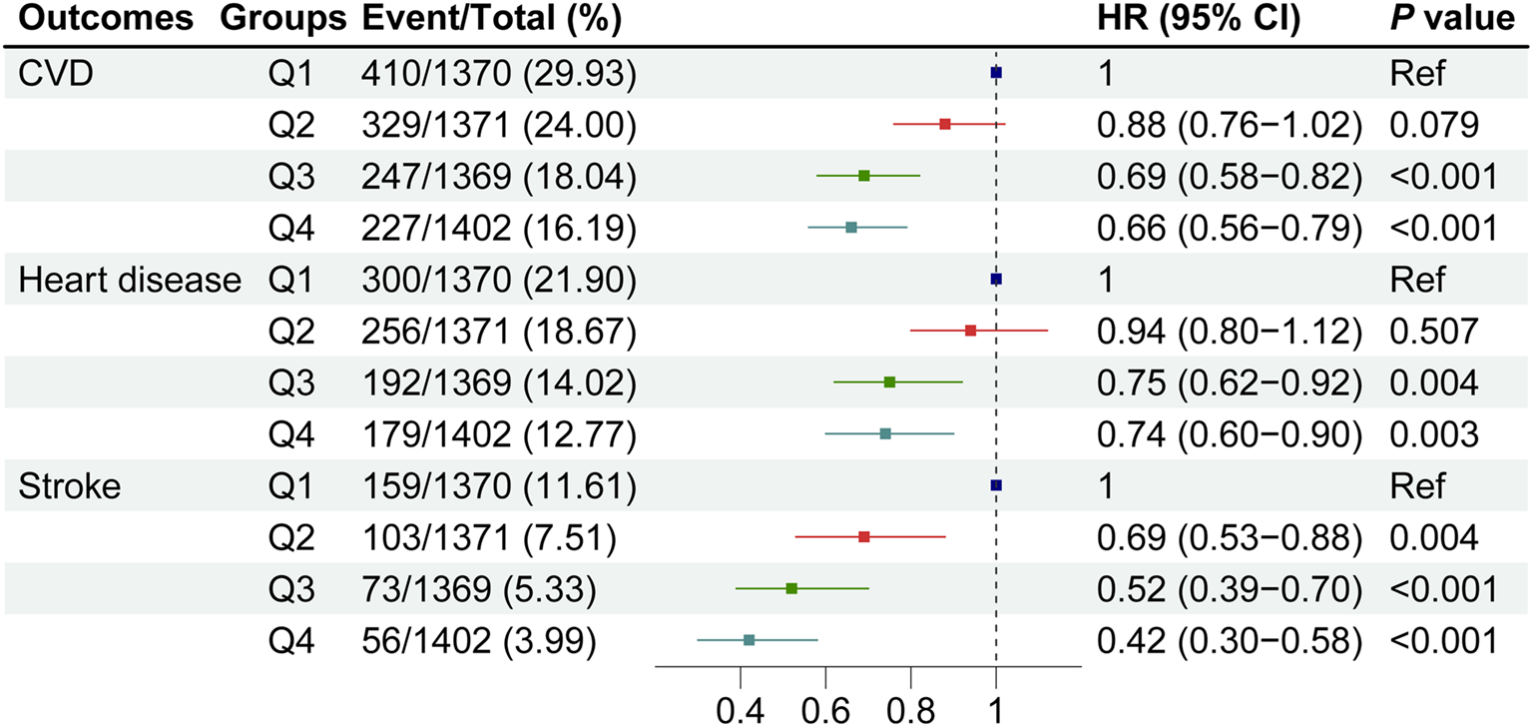
Multivariate-adjusted hazard ratios (95% confidence intervals) of estimated glucose disposal rate (quartile 1 − 4) for cardiovascular diseases in Model 3

As shown in supplementary file 1, Figure S2 − 4, Kaplan–Meier survival curves indicated that individuals with a higher eGDR had a lower cumulative incidence of CVD, heart disease, and stroke. Compared with the Q1 of eGDR, the adjusted HRs (95% CIs) of Q2 − 4 were: 0.88 (0.76 − 1.02), 0.69 (0.58 − 0.82), and 0.66 (0.56 − 0.79) for CVD; 0.94 (0.80 − 1.12), 0.75 (0.62 − 0.92), and 0.74 (0.60 − 0.90) for heart disease; 0.69 (0.53 − 0.88), 0.52 (0.39 − 0.70), and 0.42 (0.30 − 0.58) for stroke (Table 2 and Fig. 2).

**Table 2:** Multivariate-adjusted hazard ratios (95% confidence intervals) of estimated glucose disposal rate for cardiovascular diseases

eGDR	Total N	No. of events (Incident rate^a^)	Model 1		Model 2		Model 3	
			HR (95% CI)	*P* value	HR (95% CI)	*P* value	HR (95% CI)	*P* value
Cardiovascular diseases								
Continues								
Per SD increase	5512	1213 (33.25)	0.76 (0.72 − 0.80)	< 0.001	0.78 (0.74 − 0.83)	< 0.001	0.83 (0.78 − 0.89)	< 0.001
Quartiles								
Q1	1370	410 (46.29)	Ref		Ref		Ref	
Q2	1371	329 (36.55)	0.79 (0.68 − 0.92)	0.002	0.82 (0.71 − 0.95)	0.007	0.88 (0.76 − 1.02)	0.079
Q3	1369	247 (26.82)	0.56 (0.48 − 0.65)	< 0.001	0.62 (0.52 − 0.72)	< 0.001	0.69 (0.58 − 0.82)	< 0.001
Q4	1402	227 (24.11)	0.52 (0.44 − 0.61)	< 0.001	0.56 (0.47 − 0.66)	< 0.001	0.66 (0.56 − 0.79)	< 0.001
* P* for trend				< 0.001		< 0.001		< 0.001
Heart disease								
Continues								
Per SD increase	5512	927 (25.23)	0.79 (0.74 − 0.84)	< 0.001	0.82 (0.77 − 0.87)	< 0.001	0.87 (0.81 − 0.94)	< 0.001
Quartiles								
Q1	1370	300 (33.45)	Ref		Ref		Ref	
Q2	1371	256 (28.21)	0.85 (0.72 − 1.00)	0.052	0.88 (0.75 − 1.04)	0.140	0.94 (0.80 − 1.12)	0.507
Q3	1369	192 (20.74)	0.60 (0.50 − 0.72)	< 0.001	0.67 (0.56 − 0.80)	< 0.001	0.75 (0.62 − 0.92)	0.004
Q4	1402	179 (18.96)	0.57 (0.47 − 0.68)	< 0.001	0.62 (0.51 − 0.75)	< 0.001	0.74 (0.60 − 0.90)	0.003
* P* for trend				< 0.001		< 0.001		< 0.001
Stroke								
Continues								
Per SD increase	5512	391 (10.25)	0.65 (0.59 − 0.72)	< 0.001	0.67 (0.61 − 0.74)	< 0.001	0.70 (0.63 − 0.78)	< 0.001
Quartiles								
Q1	1370	159 (16.88)	Ref		Ref		Ref	
Q2	1371	103 (10.87)	0.64 (0.50 − 0.82)	< 0.001	0.65 (0.51 − 0.83)	0.001	0.69 (0.53 − 0.88)	0.004
Q3	1369	73 (7.67)	0.44 (0.33 − 0.58)	< 0.001	0.48 (0.36 − 0.63)	< 0.001	0.52 (0.39 − 0.70)	< 0.001
Q4	1402	56 (5.74)	0.34 (0.25 − 0.47)	< 0.001	0.36 (0.26 − 0.49)	< 0.001	0.42 (0.30 − 0.58)	< 0.001
* P* for trend				< 0.001		< 0.001		< 0.001

Model 1: unadjusted

Model 2: adjusted for age, sex, rural residence, marital status, education, smoking, and alcohol consumption status

Model 3: model 2 + further adjusted for region, TC, HDL, TG, LDL, BUN, UA, hsCRP, hemoglobin, chronic kidney disease, and obesity

*BUN* blood urea nitrogen, *CI* confidence interval, *eGDR* estimated glucose disposal rate, *HDL* high density lipoprotein, *HR* hazard ratio, *hsCRP* high-sensitivity C-reactive protein, *LDL* low density lipoprotein, *Ref* reference, *TC* total cholesterol, *TG* triglyceride, *UA* uric acid

^a^Incident rate was presented as per 1000 person-years of follow-up

### Mediation analyses

In mediation analyses (supplementary file 1, Figure S5-6), obesity played a significant mediating role in associations of the eGDR with incident CVD, heart disease, and stroke. In the unadjusted models, obesity accounted for 8.1%, and 11.4% of the associations of eGDR with incident CVD and stroke, respectively. After fully adjusted covariates, the mediated proportions through obesity were 14.0% and 17.6% for the effects of eGDR on incidence of CVD and heart disease, respectively. The mediation effects of eGDR on associations between eGDR and stroke were not significant in either unadjusted or adjusted models (supplementary file 1, Figure S7).

### Subgroup analyses

**Fig. 3:**
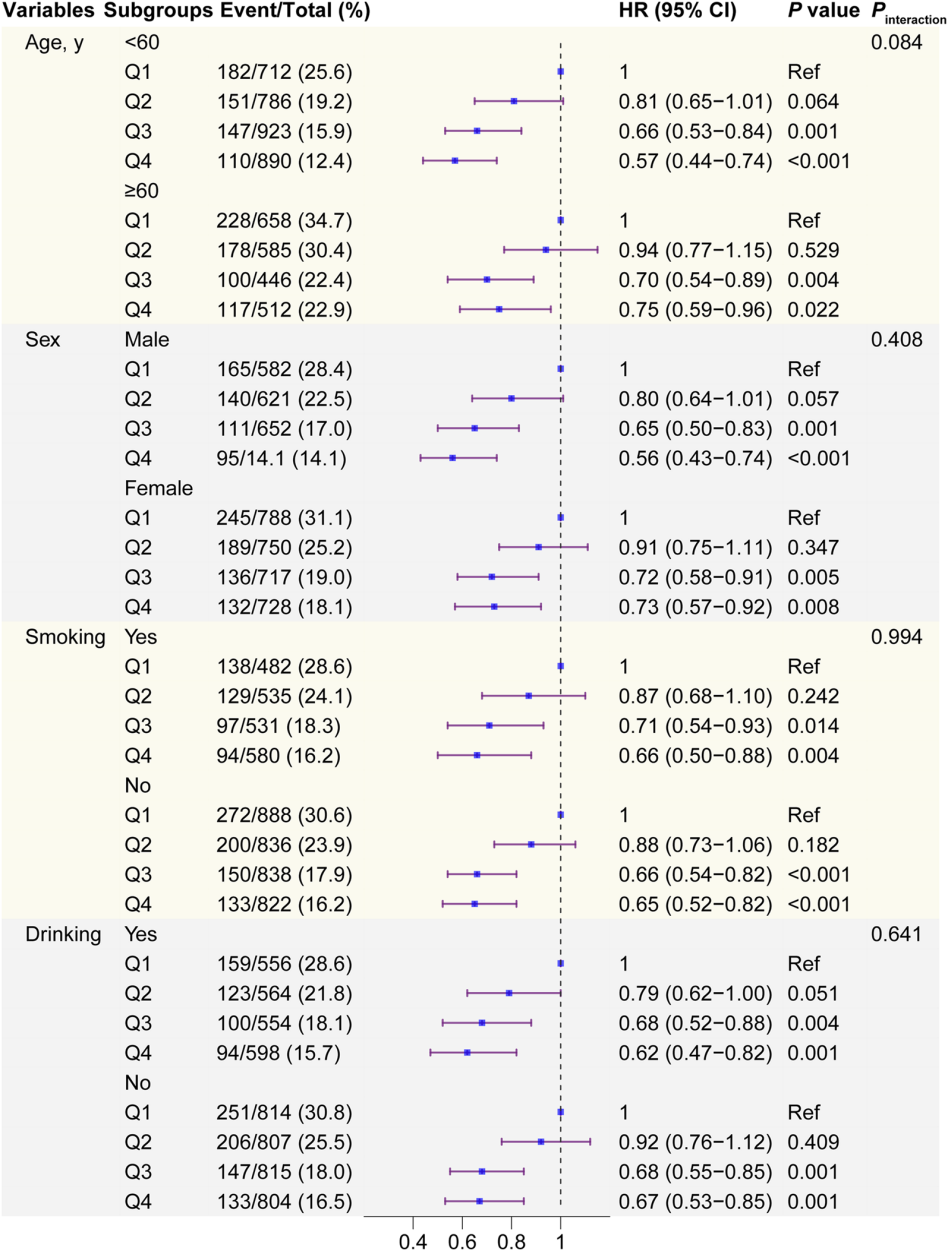
Subgroup and interaction analyses among the quartile 1 − 4 and CVD across various subgroups

Stratified analyses were used to assess whether the associations between eGDR (both continuous and categorical) and incidence of CVD were modified by pre-specified subgroups. The relationship between eGDR and the incidence of CVD in most subgroups was consistent with the main results (Table 3). The predictive performance of eGDR on incidence of CVD only modified by smoking subgroups (*P* for interaction = 0.012). No significant interactions on the associations between quartles of eGDR and incidence of different endpoints were observed among these subgroups (Fig. 3, and supplementary file 1, Figure S8–S9).

**Table 3:** Subgroup analysis of the association between estimated glucose disposal rate and cardiovascular diseases

Variables^a^	Cardiovascular diseases		Heart disease		Stroke	
	HR (95% CI)	*P*_interaction_	HR (95% CI)	*P*_interaction_	HR (95% CI)	*P*_interaction_
Age, years		0.189		0.214		0.223
< 60	0.80 (0.73 − 0.88)***		0.84 (0.76 − 0.93)**		0.65 (0.55 − 0.76)***	
≥ 60	0.86 (0.79 − 0.94)***		0.90 (0.82 − 0.99)*		0.75 (0.65 − 0.87)***	
Sex		0.085		0.330		0.309
Male	0.79 (0.72 − 0.87)***		0.83 (0.75 − 0.93)**		0.66 (0.57 − 0.77)***	
Female	0.87 (0.79 − 0.94)***		0.90 (0.82 − 0.98)*		0.74 (0.63 − 0.87)***	
Smoking		0.012		0.595		0.513
Yes	0.84 (0.76 − 0.93)**		0.89 (0.79 − 1.01)		0.67 (0.57 − 0.79)***	
No	0.82 (0.76 − 0.89)***		0.86 (0.79 − 0.94)**		0.71 (0.61 − 0.82)***	
Drinking		0.466		0.618		0.682
Yes	0.82 (0.75 − 0.91)***		0.86 (0.76 − 0.96)**		0.73 (0.62 − 0.85)***	
No	0.83 (0.77 − 0.90)***		0.87 (0.80 − 0.96)**		0.67 (0.58 − 0.78)***	

Adjusted variables included age, sex, rural residence, marital status, education, smoking, alcohol consumption status, region, TC, HDL, TG, LDL, BUN, UA, hsCRP, hemoglobin, chronic kidney disease, and obesity

*BUN* blood urea nitrogen, *CI* confidence interval, *eGDR* estimated glucose disposal rate, *HDL* high density lipoprotein, *HR* hazard ratio, *hsCRP* high-sensitivity C-reactive protein, *LDL* low density lipoprotein, *Ref* reference, *TC* total cholesterol, *TG* triglyceride, *UA* uric acid

^a^Per SD increase

*P < 0.05, **P < 0.01, and ***P < 0.001

### Sensitivity analyses

In the sensitivity analyses, the results did not materially change when only included individuals with normal glucose status (supplementary file 1, Table S2). Similar results were observed when we recalculated the eGDR using the redefined hypertension (≥ 130/80 mm Hg) (supplementary file 1, Table S3). Consistent results were demonstrated after excluding participants who suffered frm CVD during or before wave 2 (supplementary file 1, Table S4). The results were largely unchanged when we excluded diabetic patients defined according to the measures of FBG and HbA1c (supplementary file 1, Table S5).

### Incremental predictive performance of eGDR in the incident CVD

**Fig. 4:**
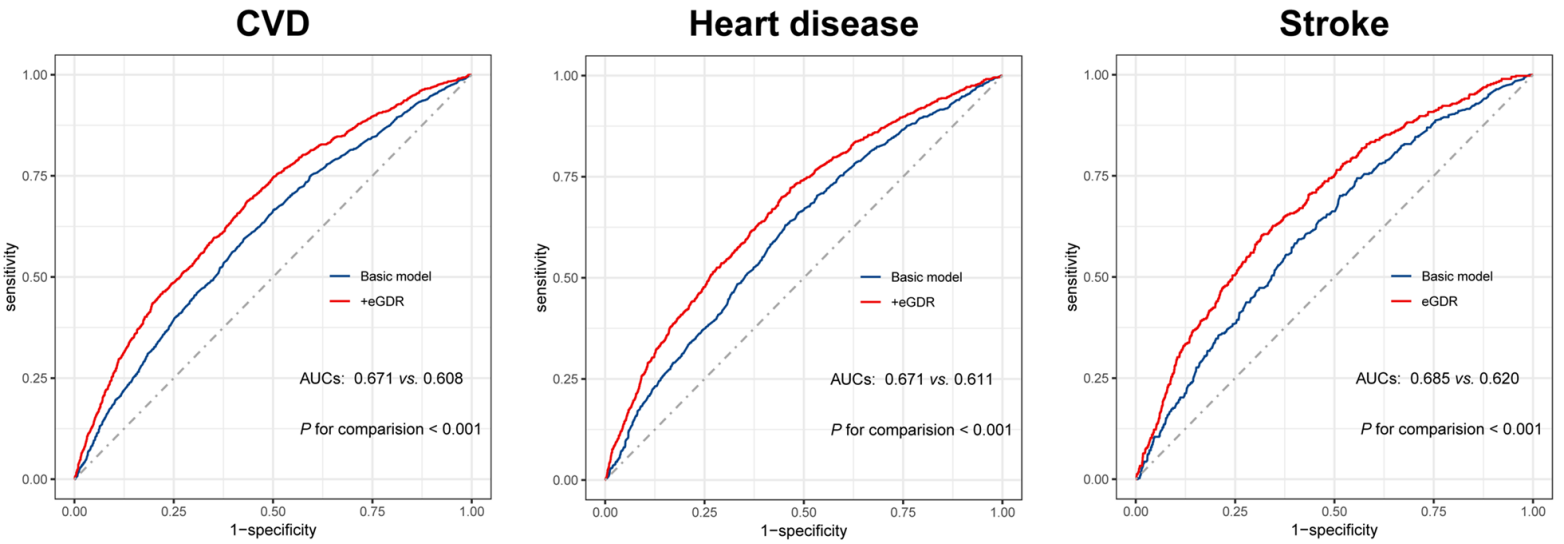
The receiver operating characteristic curves of the eGDR as an IR marker to predict MACCEs. The basic model adjusted age, sex, rural residence, marital status, education, smoking, alcohol consumption status, region, TC, HDL, TG, LDL, BUN, UA, hsCRP, hemoglobin, chronic kidney disease, and obesity

Based on the Model 3, the basic models were constructed (including age, sex, rural residence, marital status, education, smoking, alcohol consumption status, region, TC, HDL, TG, LDL, BUN, UA, hsCRP, hemoglobin, chronic kidney disease, and obesity). Adding the eGDR significantly optimized the predictive ability of the basic model for CVD (*C*-statistics: 0.671 *vs.* 0.608, *P* < 0.001), heart disease (*C*-statistics: 0.671 *vs.* 0.611, *P* < 0.001), and stroke (*C*-statistics: 0.685 *vs.* 0.620 *P* < 0.001) (Table 4 and Fig. 4). Moreover, all the NRI and IDI for CVD, heart disease, and stroke were significant (all *P* < 0.001) (Table 4). Despite the inclusion of hypertension in the basic model, which did enhance its predictive capability for cardiovascular outcomes, it remains inferior in strength compared to our capacity to incorporate eGDR.

**Table 4:** Improvement in discrimination and risk reclassification for cardiovascular diseases after adding estimated glucose disposal

Model	*C*-statistic (95% CI)	*P* value	NRI (95% CI)	*P* value	IDI (95% CI)	*P* value
CVD						
Basic model	0.608 (0.590 − 0.626)	Ref	Ref		Ref	
+ hypertension	0.628 (0.611 − 0.646)	0.019	0.050 (0.018 − 0.082)	0.003	0.007 (0.017 − 0.012)	< 0.001
+ eGDR	0.671 (0.654 − 0.688)	< 0.001	0.167 (0.130 − 0.210)	< 0.001	0.041 (0.035 − 0.047)	< 0.001
Heart disease						
Basic model	0.611 (0.592 − 0.631)	Ref	Ref		Ref	
+ hypertension	0.623 (0.603 − 0.642)	0.019	0.043 (0.014 − 0.072)	0.003	0.005 (0.003 − 0.007)	< 0.001
+ eGDR	0.671 (0.652 − 0.690)	< 0.001	0.138 (0.096 − 0.179)	< 0.001	0.034 (0.028 − 0.039)	< 0.001
Stroke						
Basic model	0.620 (0.592 − 0.648)	Ref	Ref		Ref	
+ hypertension	0.659 (0.633 − 0.686)	< 0.001	0.009 (− 0.002 − 0.020)	0.116	0.010 (0.007 − 0.013)	< 0.001
+ eGDR	0.685 (0.658 − 0.712)	< 0.001	0.038 (0.015 − 0.061)	0.001	0.020 (0.015 − 0.024)	< 0.001

The basic model included age, sex, rural residence, marital status, education, smoking, alcohol consumption status, region, TC, HDL, TG, LDL, BUN, UA, hsCRP, hemoglobin, chronic kidney disease, and obesity

*BUN* blood urea nitrogen, *CI* confidence interval, *CVD* cardiovascular diseases, *eGDR* estimated glucose disposal rate, *HDL* high density lipoprotein, *HR* hazard ratio, *hsCRP* high-sensitivity C-reactive protein, *IDI* integrated discrimination improvement, *LDL* low density lipoprotein, *NRI* net reclassification improvement, *Ref* reference, *TC* total cholesterol, *TG* triglyceride, *UA* uric acid

## Discussion

The predictive value of eGDR for incident CVD among individuals without diabetes is being examined for the first time in our study. The main findings could be summarized as: (1) lower eGDR was significantly related to higher risk of incident CVD (including heart disease and stroke); (2) these association were linear and independent of age, sex, smoking and alcohol consumption status; (3) obesity partly mediated the relationship between eGDR and CVD; (4) the eGDR significantly enhanced the predictive power of basic models for adverse cardiovascular outcomes.

IR has been demonstrated to be associated with diabetes, impaired lipid metabolism, and the elevated BP, which are the major risk factors of incident CVD [5, 30, 31]. Previous studies suggested that HOMA-IR, as a reliable surrogate marker of IR, is associated with higher risk of incident CVD in general populations or adults with or without diabetes [32–35]. The association between another surrogate indicator, triglyceride-glucose (TyG index), and CVD has been widely examined in the previous studies, and the results consistently showed elevated TyG index positively contributed to increased risk of CVD or severity of coronary heart disease [36–38]. Taking together, these results suggest IR may be a novel and promising biomarker to predictive the incident CVD. However, the calculation of HOMA-IR is based on the measures of fasting blood glucose and insulin, which severely limits the popularity of this technique, because fasting insulin is not routinely tested for nondiabetic patients. Moreover, many factors, such as using of insulin, insulin sensitizers, and insulin secretagogues, could disturb the measurement of HOMA-IR, resulting in a misclassification [39, 40]. Similarly, the sensitivity and specificity of TyG index seems to be not satisfying in some conditions [41, 42]. As anticipated, our findings revealed that the predictive performance of eGDR for CVD incidence was markedly superior to that of the TyG index in non-diabetic participants. This could be attributed to the integration of clinical and laboratory data in the calculation of eGDR, thereby offering a more comprehensive assessment of insulin resistance. Considered the invasiveness and expenditure of the traditional methods assessed the status of IR, the calculation of eGDR only based on participants’ WC, HbA1c, and presence of hypertension, makes it more suitable large-scale clinical applications. More importantly, eGDR has a similar accuracy with HIEG clamp in assessing the status of IR [6, 29], exhibited a good ability in predicting the incidence of CVD, and the explained attributable relative risk of CVD may be at least partly contributed to each item in the formular of eGDR. It’s noteworthy that in individuals without diabetes, WC and hypertension exert a more significant influence on eGDR values compared to HbA1c. This is because HbA1c levels are relatively low in non-diabetic patients in our study. However, it’s important to highlight that these three variables—WC, hypertension, and HbA1c—contribute to eGDR to a similar extent.

Considerable studies have suggested that decreased eGDR is tightly related with high risk of adverse cardiovascular events. Zabala et al. found a low eGDR increased greatly the risk of stroke and mortality among patients with type 2 diabetes [6]. Another study of 774 subjects with type 1 diabetes over a 10-year follow-up showed that per 1.0-SD increases in eGDR is associated with 44% (HR: 0.56, 95% CI: 0.39 − 0.80) lower risk of incident major cardiovascular events and 37% lower risk of coronary artery disease (HR: 0.63, 95% CI: 0.42 − 0.96), which is similar with our results. Similarly, the China National Stroke Registry III study demonstrated that eGDR was a reliable predictor for functional outcomes among patients with acute ischemic stroke [42]. More currently, a study indicated that eGDR was independently associated with all-cause mortality in diabetic patients without diabetic kidney disease (HR: 1.214, 95% CI: 1.072 − 1.375) [7]. However, these studies tended to only include specific populations, particular participants with type 1 or 2 diabetes, a well-recognized risk factor for incident CVD, therefore the detrimental effects of eGDR on cardiovascular outcomes may be exaggerated [43]. In a recent study, the findings indicated that eGDR is associated with a heightened risk of cardiovascular disease (CVD) in the general population, and this association remains consistent regardless of diabetic status. Interestingly, eGDR appears to exhibit greater sensitivity in predicting CVD among non-diabetic individuals (Q4 vs. Q1: HR: 0.59, 95% CI: 0.50–0.69 in non-diabetic individuals; HR: 0.75, 95% CI: 0.56–0.96 in diabetic individuals) [16]. This observation aligns with our sensitivity analysis conducted among participants with normal glucose status, wherein prediabetic individuals were further excluded. Prior research also suggests that diabetic individuals may exhibit reduced sensitivity to predictive indicators due to the presence of additional risk factors, a phenomenon commonly referred to as the ceiling effect [18, 44]. Moreover, the identification of risk factors in non-diabetic populations could prompt earlier intervention efforts, thereby carrying significant implications for disease burden reduction. Based on the reasons mentioned above, we investigated the association of eGDR with incident CVD among individuals free of diabetes based on a national cohort. Our robust results extended the understanding on the association of IR with risk of CVD.

Comparted with the previous studies, it is the first time to assess the predictive performance of eGDR for incident CVD among nondiabetic individuals through adding it in the basic models. Our results suggested that the eGDR significantly enhanced the predictive power of basic models for incident CVD, heart disease, and stroke, which is expected to update the CVD prediction scoring system in the future. In addition, given that hyperglycemia caused by IR may contribute to obesity, and ultimately result in CVD, we investigated the role of obesity in the association between eGDR and incident CVD by performing mediation analysis. The results showed the obesity partly mediated the relationship, therefore controlling body mass may alleviate the unfavorable influencing of IR on circulatory system.

Despite the exact biological mechanisms on how IR contributes to CVD is not completely understood, several plausible explanations have been proposed. Firstly, IR contributes to the progression of atherosclerosis: elevated insulin levels can inhibit the release of nitric oxide (NO) by activating glucocorticoid-regulated kinase 1. This, in turn, can lead to the deposition of matrix proteins and fibrosis, ultimately directly or indirectly causing a reduction in vascular dilation function and atherosclerosis [45]. Secondly, IR-induced lipid and glucose metabolism impairment: IR can activate protein kinase C and the nuclear factor κB pathway, leading to excessive production of reactive oxygen species. These pathways trigger inflammatory responses and endothelial damage, which can ultimately trigger cardiovascular events [46]. Thirdly, inappropriately activated RAAS leads to fluid retention and hypertension [4, 10]. Lastly, the effect of insulin on thrombosis and platelet aggregation [47]: IR may cause a procoagulant tendency by reducing hemostatic markers levels in circulation [48].

There are several limitations should be noted. Firstly, due to the nature of observational studies, we are unable to establish causality, and there may even be the possibility of reverse causality. However, this seems to be minimal because even when we exclude individuals who experienced the endpoint within the first two years, the results remain stable. Secondly, although our model adjusts for many covariates, residual confounding still cannot be completely eliminated. This is a common issue in observational studies. Thirdly, the endpoint events in this study are self-reported by participants based on diagnoses from physicians, which may introduce recall bias, leading to inevitable misclassification. However, this is widely accepted in cohort studies, and it has been demonstrated that its impact is minimal [38]. Fourthly, while the determination of HbA1c is typically conducted using standard methods, it’s important to note that certain unavoidable disease states and conditions may influence its accuracy. These include factors such as iron deficiency anemia, the administration of erythropoietin, and splenectomy [49]. Finally, our study only included Chinese individuals aged 45 and above, which may affect the generalizability of our conclusions. Therefore, further studies are urgently needed to confirm our results through broader population samples and diverse demographics.

## Conclusion

Our study shows that eGDR, a reliable surrogate marker of IR, is a strong predictor for incident CVD among nondiabetic individuals. Those individuals with lower eGDR levels had higher risk of CVD in the future. Incorporating the eGDR into the basic risk model significantly enhances the predictive performance for CVD. These findings may guide preventive measures and lowering the burden of CVD by improving risk assessment in individuals without diabetes.

### Supplementary Information

Supplementry file1 (DOCX 2350 kb)

## Data Availability

The data supporting the findings of this study are available the CHARLS website (http://charls.pku.edu.cn/en).

## Abbreviations

BMI: Body mass index
BP: Blood pressure
BUN: Blood urea nitrogen
CHARLS: China health and retirement longitudinal study
CI: Confidence interval
CVD: Cardiovascular diseases
eGDR: Estimated glucose disposal rate
FBG: Fasting blood glucose
HbA1c: Glycosylated hemoglobin A1c
HDL: High-density lipoprotein
HIEG: Hyperinsulinemic- euglycemic
HOMA-IR: Homeostasis model assessment for insulin resistance
HR: Hazard ratio
hsCRP: High-sensitivity C-reactive protein
IDI: Integrated discrimination improvement
LDL: Low-density lipoprotein
NRI: Net reclassification improvement
Q: Quartiles
RAAS: Renin–angiotensin–aldosterone system
RCS: Restricted cubic spline
TC: Total cholesterol
TG: Triglyceride
TyG: Triglyceride-glucose
UA: Uric acid
WC: Waist circumference

## References

[1] Roth GA, Mensah GA, Johnson CO, Addolorato G, Ammirati E, Baddour LM, Barengo NC, Beaton AZ, Benjamin EJ, Benziger CP (2020). Global burden of cardiovascular diseases and risk factors, 1990–2019: update from the GBD 2019 study. J Am Coll Cardiol.

[2] Zhao D, Liu J, Wang M, Zhang X, Zhou M (2019). Epidemiology of cardiovascular disease in China: current features and implications. Nat Rev Cardiol.

[3] GBD 2017 Causes of Death Collaborators (2018). Global, regional, and national age-sex-specific mortality for 282 causes of death in 195 countries and territories, 1980–2017: a systematic analysis for the Global Burden of Disease Study 2017. Lancet.

[4] Zhang Z, Zhao L, Lu Y, Meng X, Zhou X (2023). Association between non-insulin-based insulin resistance indices and cardiovascular events in patients undergoing percutaneous coronary intervention: a retrospective study. Cardiovasc Diabetol.

[5] Jin A, Wang S, Li J, Wang M, Lin J, Li H, Meng X, Wang Y, Pan Y (2023). Mediation of systemic inflammation on insulin resistance and prognosis of nondiabetic patients with ischemic stroke. Stroke.

[6] Zabala A, Darsalia V, Lind M, Svensson AM, Franzen S, Eliasson B, Patrone C, Jonsson M, Nystrom T (2021). Estimated glucose disposal rate and risk of stroke and mortality in type 2 diabetes: a nationwide cohort study. Cardiovasc Diabetol.

[7] Penno G, Solini A, Orsi E, Bonora E, Fondelli C, Trevisan R, Vedovato M, Cavalot F, Zerbini G, Lamacchia O (2021). Insulin resistance, diabetic kidney disease, and all-cause mortality in individuals with type 2 diabetes: a prospective cohort study. BMC Med.

[8] Ye Z, Xu Y, Tang L, Wu M, Wu B, Zhu T, Wang J (2023). Predicting long-term prognosis after percutaneous coronary intervention in patients with new onset ST-elevation myocardial infarction: development and external validation of a nomogram model. Cardiovasc Diabetol.

[9] Bornfeldt KE, Tabas I (2011). Insulin resistance, hyperglycemia, and atherosclerosis. Cell Metab.

[10] Jia G, Whaley-Connell A, Sowers JR (2018). Diabetic cardiomyopathy: a hyperglycaemia- and insulin-resistance-induced heart disease. Diabetologia.

[11] Bonora E, Targher G, Alberiche M, Bonadonna RC, Saggiani F, Zenere MB, Monauni T, Muggeo M (2000). Homeostasis model assessment closely mirrors the glucose clamp technique in the assessment of insulin sensitivity: studies in subjects with various degrees of glucose tolerance and insulin sensitivity. Diabetes Care.

[12] Li H, Zuo Y, Qian F, Chen S, Tian X, Wang P, Li X, Guo X, Wu S, Wang A (2022). Triglyceride-glucose index variability and incident cardiovascular disease: a prospective cohort study. Cardiovasc Diabetol.

[13] Nyström T, Holzmann MJ, Eliasson B, Svensson AM, Sartipy U (2018). Estimated glucose disposal rate predicts mortality in adults with type 1 diabetes. Diabetes Obes Metab.

[14] Olson JC, Erbey JR, Williams KV, Becker DJ, Edmundowicz D, Kelsey SF, Tyrrell KS, Orchard TJ (2002). Subclinical atherosclerosis and estimated glucose disposal rate as predictors of mortality in type 1 diabetes. Ann Epidemiol.

[15] Garofolo M, Gualdani E, Scarale MG, Bianchi C, Aragona M, Campi F, Lucchesi D, Daniele G, Miccoli R, Francesconi P (2020). Insulin resistance and risk of major vascular events and all-cause mortality in type 1 diabetes: a 10-year follow-up study. Diabetes Care.

[16] Ren X, Jiang M, Han L, Zheng X (2022). Estimated glucose disposal rate and risk of cardiovascular disease: evidence from the China health and retirement longitudinal study. BMC Geriatr.

[17] Dal Canto E, Ceriello A, Rydén L, Ferrini M, Hansen TB, Schnell O, Standl E, Beulens JW (2019). Diabetes as a cardiovascular risk factor: an overview of global trends of macro and micro vascular complications. Eur J Prev Cardiol.

[18] Zhang P, Guo D, Xu B, Huang C, Yang S, Wang W, Liu W, Deng Y, Li K, Liu D (2022). Association of serum 25-hydroxyvitamin D with cardiovascular outcomes and all-cause mortality in individuals with prediabetes and diabetes: results from the UK biobank prospective cohort study. Diabetes Care.

[19] Harding JL, Pavkov ME, Magliano DJ, Shaw JE, Gregg EW (2019). Global trends in diabetes complications: a review of current evidence. Diabetologia.

[20] Kahn SE, Hull RL, Utzschneider KM (2006). Mechanisms linking obesity to insulin resistance and type 2 diabetes. Nature.

[21] Zhao Y, Hu Y, Smith JP, Strauss J, Yang G (2014). Cohort profile: the China health and retirement longitudinal study (CHARLS). Int J Epidemiol.

[22] Williams B, Mancia G, Spiering W, Agabiti Rosei E, Azizi M, Burnier M, Clement DL, Coca A, de Simone G, Dominiczak A (2018). 2018 ESC/ESH guidelines for the management of arterial hypertension. Eur Heart J.

[23] Yu J, Yi Q, Chen G, Hou L, Liu Q, Xu Y, Qiu Y, Song P (2022). The visceral adiposity index and risk of type 2 diabetes mellitus in China: a national cohort analysis. Diabetes Metab Res Rev.

[24] Zheng X, Han L, Shen S (2022). Hypertension, remnant cholesterol and cardiovascular disease: evidence from the China health and retirement longitudinal study. J Hypertens.

[25] Lin L, Wang HH, Liu Y, Lu C, Chen W, Guo VY (2021). Indoor solid fuel use for heating and cooking with blood pressure and hypertension: a cross-sectional study among middle-aged and older adults in China. Indoor Air.

[26] Li H, Zheng D, Li Z, Wu Z, Feng W, Cao X, Wang J, Gao Q, Li X, Wang W (2019). Association of depressive symptoms with incident cardiovascular diseases in middle-aged and older chinese adults. JAMA Netw Open.

[27] Liang S, Chen Y, Sun X, Dong X, He G, Pu Y, Fan J, Zhong X, Chen Z, Lin Z (2023). Long-term exposure to ambient ozone and cardiovascular diseases: Evidence from two national cohort studies in China. J Adv Res.

[28] DeLong ER, DeLong DM, Clarke-Pearson DL (1988). Comparing the areas under two or more correlated receiver operating characteristic curves: a nonparametric approach. Biometrics.

[29] Liu C, Liu X, Ma X, Cheng Y, Sun Y, Zhang D, Zhao Q, Zhou Y (2022). Predictive worth of estimated glucose disposal rate: evaluation in patients with non-ST-segment elevation acute coronary syndrome and non-diabetic patients after percutaneous coronary intervention. Diabetol Metab Syndr.

[30] da Silva AA, do Carmo JM, Li X, Wang Z, Mouton AJ, Hall JE (2020). Role of hyperinsulinemia and insulin resistance in hypertension: metabolic syndrome revisited. Can J Cardiol.

[31] Semenkovich CF (2006). Insulin resistance and atherosclerosis. J Clin Invest.

[32] Bonora E, Kiechl S, Willeit J, Oberhollenzer F, Egger G, Meigs JB, Bonadonna RC, Muggeo M (2007). Insulin resistance as estimated by homeostasis model assessment predicts incident symptomatic cardiovascular disease in caucasian subjects from the general population: the Bruneck study. Diabetes Care.

[33] Bonora E, Formentini G, Calcaterra F, Lombardi S, Marini F, Zenari L, Saggiani F, Poli M, Perbellini S, Raffaelli A (2002). HOMA-estimated insulin resistance is an independent predictor of cardiovascular disease in type 2 diabetic subjects: prospective data from the verona diabetes complications study. Diabetes Care.

[34] Gast KB, Tjeerdema N, Stijnen T, Smit JW, Dekkers OM (2012). Insulin resistance and risk of incident cardiovascular events in adults without diabetes: meta-analysis. PLoS ONE.

[35] Wang T, Li M, Zeng T, Hu R, Xu Y, Xu M, Zhao Z, Chen Y, Wang S, Lin H (2022). Association between insulin resistance and cardiovascular disease risk varies according to glucose tolerance status: a nationwide prospective cohort study. Diabetes Care.

[36] Che B, Zhong C, Zhang R, Pu L, Zhao T, Zhang Y, Han L (2023). Triglyceride-glucose index and triglyceride to high-density lipoprotein cholesterol ratio as potential cardiovascular disease risk factors: an analysis of UK biobank data. Cardiovasc Diabetol.

[37] Yang X, Li K, Wen J, Yang C, Li Y, Xu G, Ma Y (2024). Association of the triglyceride glucose-body mass index with the extent of coronary artery disease in patients with acute coronary syndromes. Cardiovasc Diabetol.

[38] Cui C, Liu L, Zhang T, Fang L, Mo Z, Qi Y, Zheng J, Wang Z, Xu H, Yan H (2023). Triglyceride-glucose index, renal function and cardiovascular disease: a national cohort study. Cardiovasc Diabetol.

[39] Kozawa J, Iwahashi H, Okita K, Okauchi Y, Imagawa A, Shimomura I (2010). Insulin tolerance test predicts the effectiveness of insulin sensitizers in japanese type 2 diabetic patients. Diabetes Ther.

[40] van der Aa MP, Elst MA, van de Garde EM, van Mil EG, Knibbe CA, van der Vorst MM (2016). Long-term treatment with metformin in obese, insulin-resistant adolescents: results of a randomized double-blinded placebo-controlled trial. Nutr Diabetes.

[41] Cheng Y, Fang Z, Zhang X, Wen Y, Lu J, He S, Xu B (2023). Association between triglyceride glucose-body mass index and cardiovascular outcomes in patients undergoing percutaneous coronary intervention: a retrospective study. Cardiovasc Diabetol.

[42] Lu Z, Xiong Y, Feng X, Yang K, Gu H, Zhao X, Meng X, Wang Y (2023). Insulin resistance estimated by estimated glucose disposal rate predicts outcomes in acute ischemic stroke patients. Cardiovasc Diabetol.

[43] Li S, Liu Z, Joseph P, Hu B, Yin L, Tse LA, Rangarajan S, Wang C, Wang Y, Islam S (2022). Modifiable risk factors associated with cardiovascular disease and mortality in China: a PURE substudy. Eur Heart J.

[44] Wang T, Lu J, Su Q, Chen Y, Bi Y, Mu Y, Chen L, Hu R, Tang X, Yu X (2019). Ideal cardiovascular health metrics and major cardiovascular events in patients with prediabetes and diabetes. JAMA Cardiol.

[45] Hill MA, Jaisser F, Sowers JR (2022). Role of the vascular endothelial sodium channel activation in the genesis of pathologically increased cardiovascular stiffness. Cardiovasc Res.

[46] Chen W, Wang X, Chen J, You C, Ma L, Zhang W, Li D (2023). Household air pollution, adherence to a healthy lifestyle, and risk of cardiometabolic multimorbidity: results from the China health and retirement longitudinal study. Sci Total Environ.

[47] Brazionis L, Rowley K, Jenkins A, Itsiopoulos C, O’Dea K (2008). Plasminogen activator inhibitor-1 activity in type 2 diabetes: a different relationship with coronary heart disease and diabetic retinopathy. Arterioscler Thromb Vasc Biol.

[48] Ozkul A, Turgut ET, Akyol A, Yenisey C, Kadikoylu G, Tataroglu C, Kiylioglu N (2010). The relationship between insulin resistance and hypercoagulability in acute ischemic stroke. Eur Neurol.

[49] International Expert Committee (2009). International Expert Committee report on the role of the A1C assay in the diagnosis of diabetes. Diabetes Care.

