# Supplementary material for: Insulin resistance assessed by estimated glucose disposal rate and risk of incident cardiovascular diseases among individuals without diabetes: findings from a nationwide, population based, prospective cohort study
Source: Cardiovasc Diabetol. 2024 Jun 6;23:194. doi: 10.1186/s12933-024-02256-5 (PMC11157942; doi:10.1186/s12933-024-02256-5)
Supplement: Supplementary file 1 — Supplementry file1 (DOCX 2350 kb) [file 12933_2024_2256_MOESM1_ESM.docx]

**Insulin resistance assessed by estimated glucose disposal rate and risk of incident cardiovascular diseases among individuals without diabetes: Findings from a nationwide, population based, prospective cohort study**

**Supplemental Figures**

**Supplementary Figure 1** The flowchart of study participants.

**Supplementary Figure 2** Kaplan–Meier curves for the cumulative incidence of cardiovascular disease.

**Supplementary Figure 3** Kaplan–Meier curves for the cumulative incidence of heart disease.

**Supplementary Figure 4** Kaplan–Meier curves for the cumulative incidence of stroke.

**Supplementary Figure 5** Mediation effect of obesity between the eGDR and cardiovascular diseases.

**Supplementary Figure 6** Mediation effect of obesity between estimated glucose disposal rate and heart disease.

**Supplementary Figure 7** Mediation effect of obesity between estimated glucose disposal rate and stroke.

**Supplementary Figure 8** Subgroup analysis of HRs (95% CIs) for heart disease of estimated glucose disposal rate

**Supplementary Figure 9** Subgroup analysis of HRs (95% CIs) for heart disease of estimated glucose disposal rate

**Supplemental Tables**

**Table S1.** Baseline characteristics of participants by outcomes

**Table S2**. The association of estimated glucose disposal rate with cardiovascular diseases among participants with normal glucose status.

**Table S3**. The association of estimated glucose disposal rate (defined hypertension based on 130/80 mmHg) with cardiovascular diseases among participants.

**Table S4**. The association of estimated glucose disposal rate with cardiovascular diseases among participants after excluding individuals experienced CVD during or before Survey 2.

**Table S5**. The association of estimated glucose disposal rate with cardiovascular diseases among non-DM participants (defined DM based on FBG and HbA1c).

**Supplementary Figure 1** The flowchart of study participants.


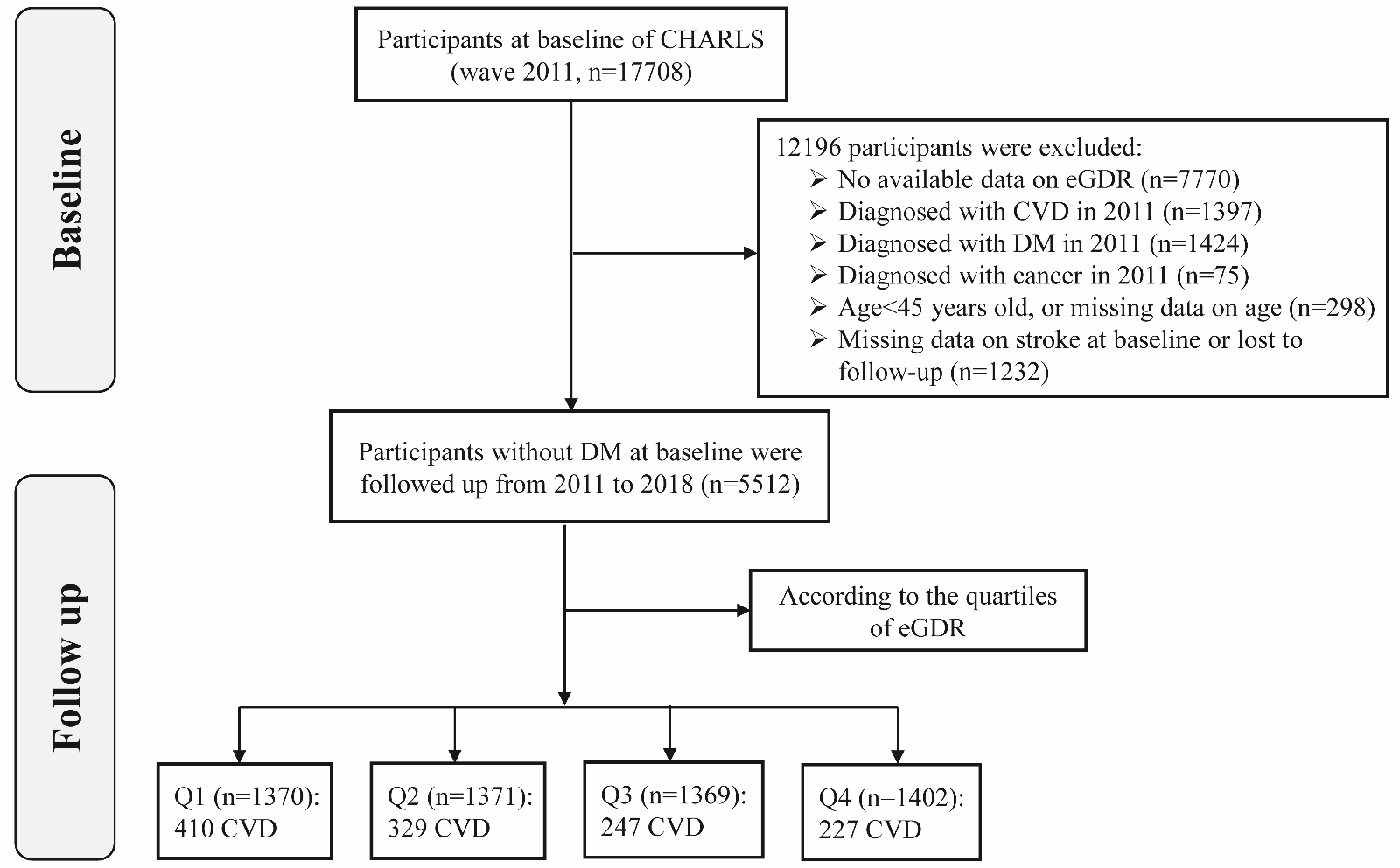


**Supplementary Figure 2** Kaplan–Meier curves for the cumulative incidence of cardiovascular disease.


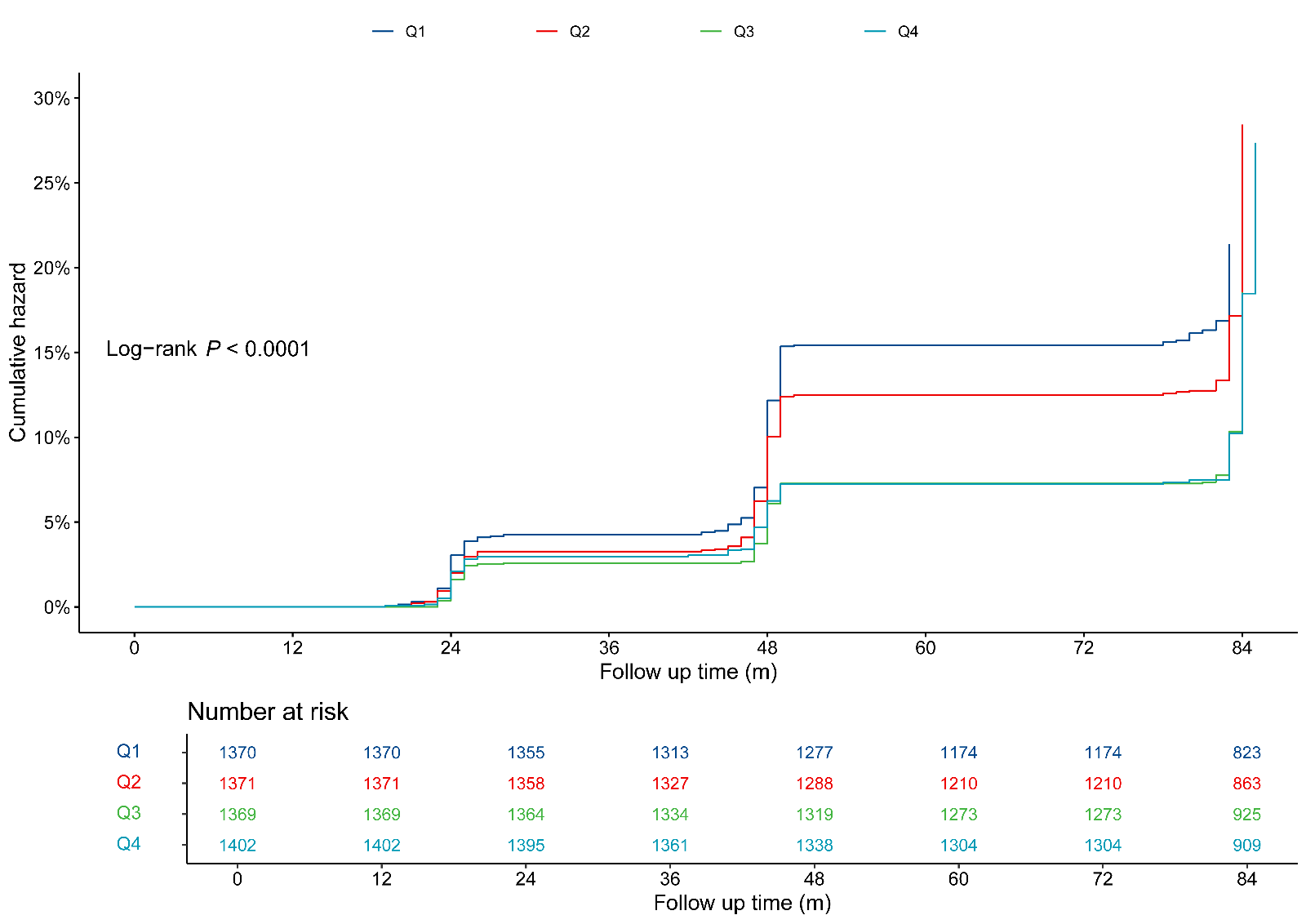


**Supplementary Figure 3** Kaplan–Meier curves for the cumulative incidence of heart disease.


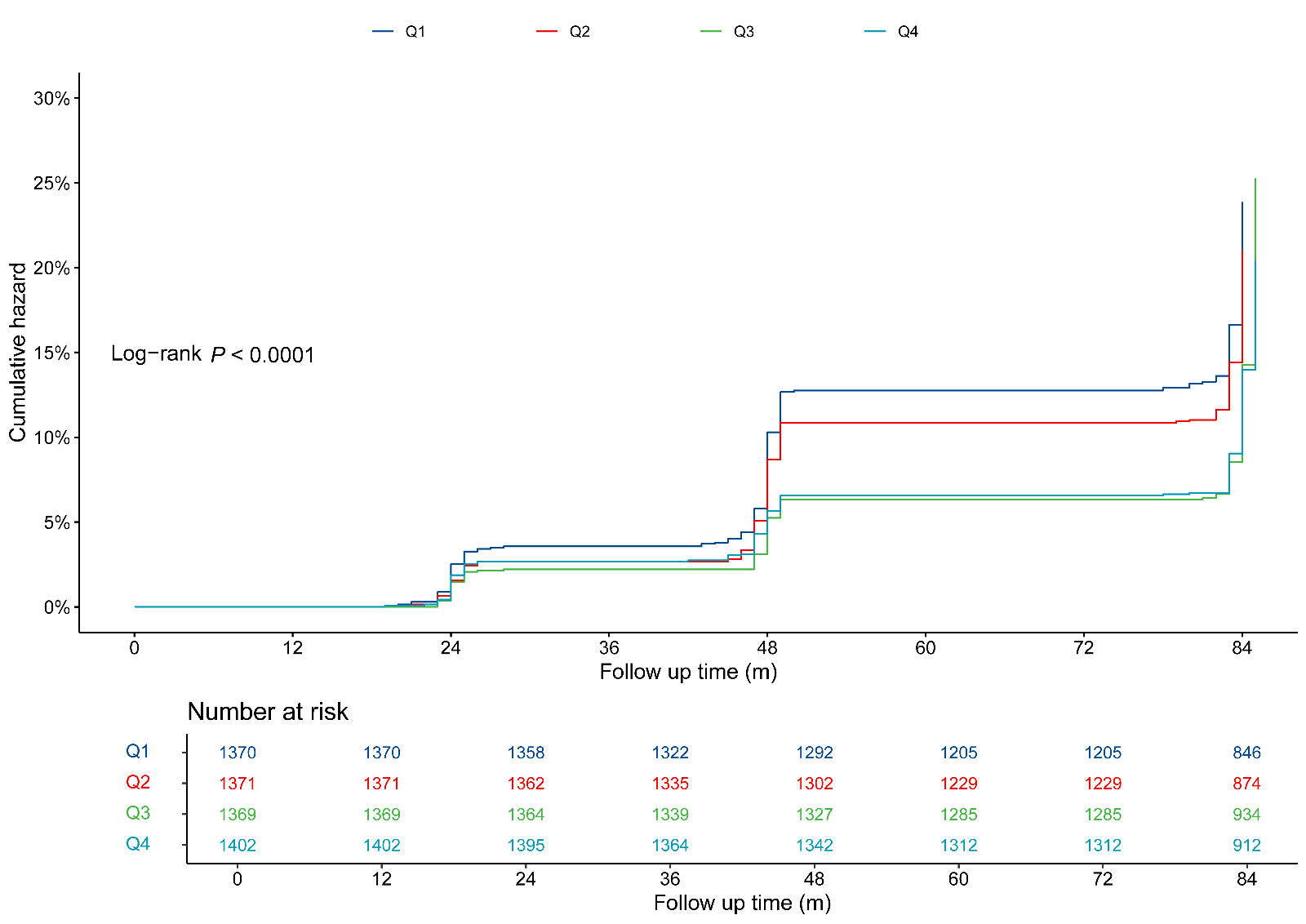


**Supplementary Figure 4** Kaplan–Meier curves for the cumulative incidence of stroke.


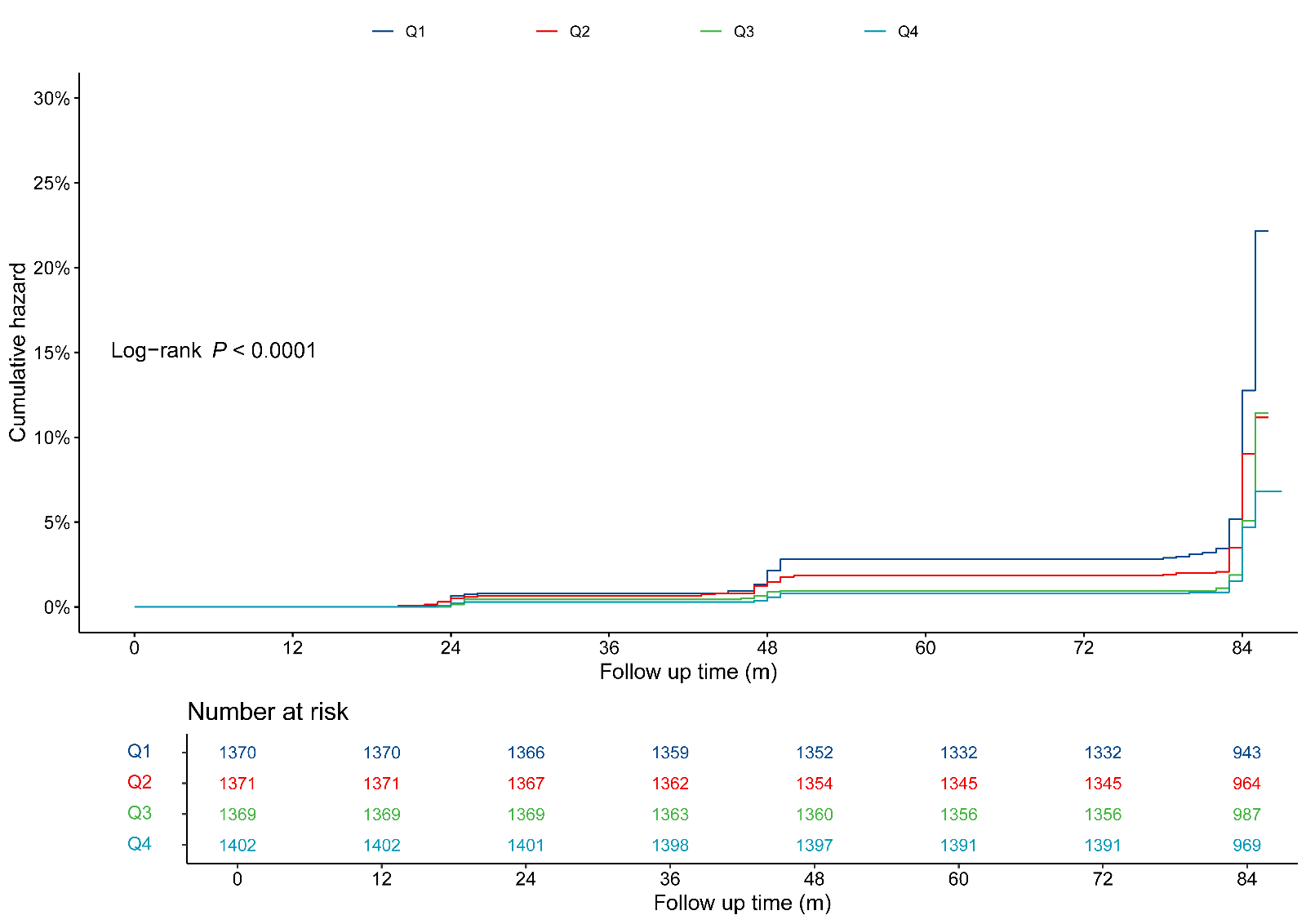


**Supplementary Figure 5** Mediation effect of obesity between the eGDR and cardiovascular diseases.


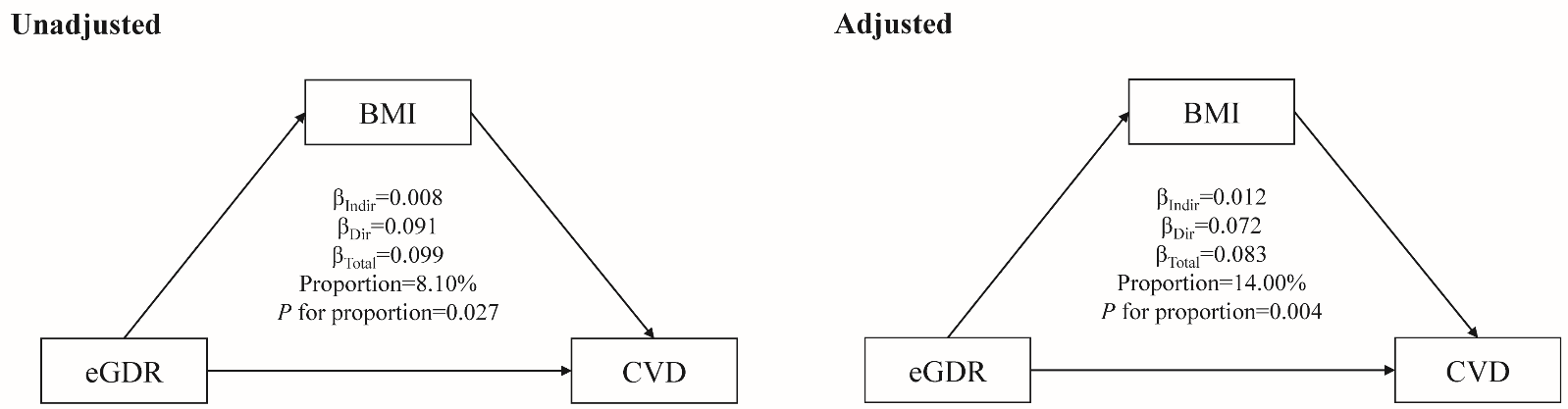


**Supplementary Figure 6** Mediation effect of obesity between estimated glucose disposal rate and heart disease.


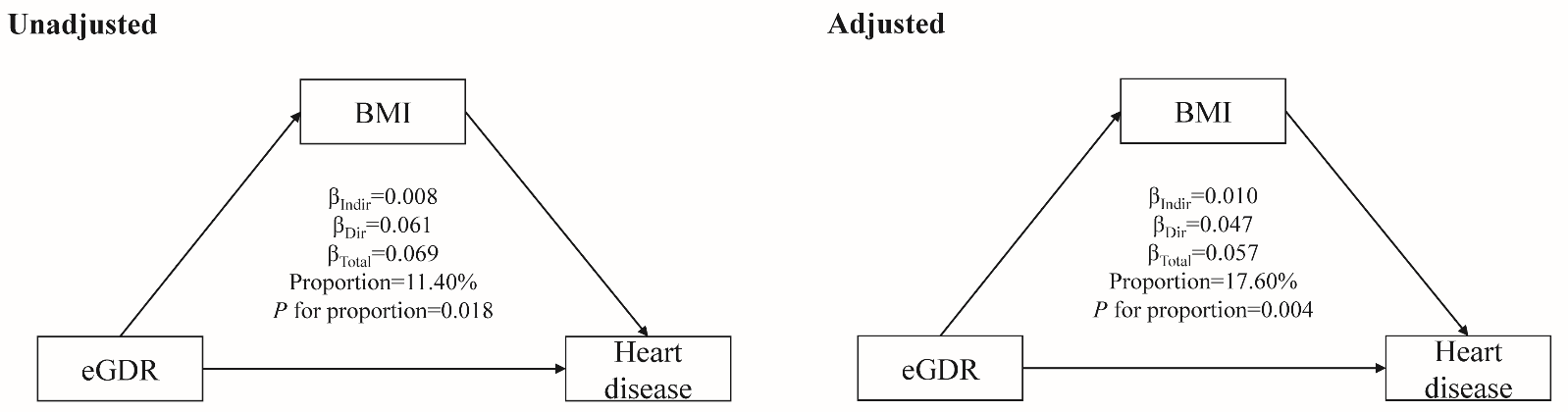


**Supplementary Figure 7** Mediation effect of obesity between estimated glucose disposal rate and stroke.


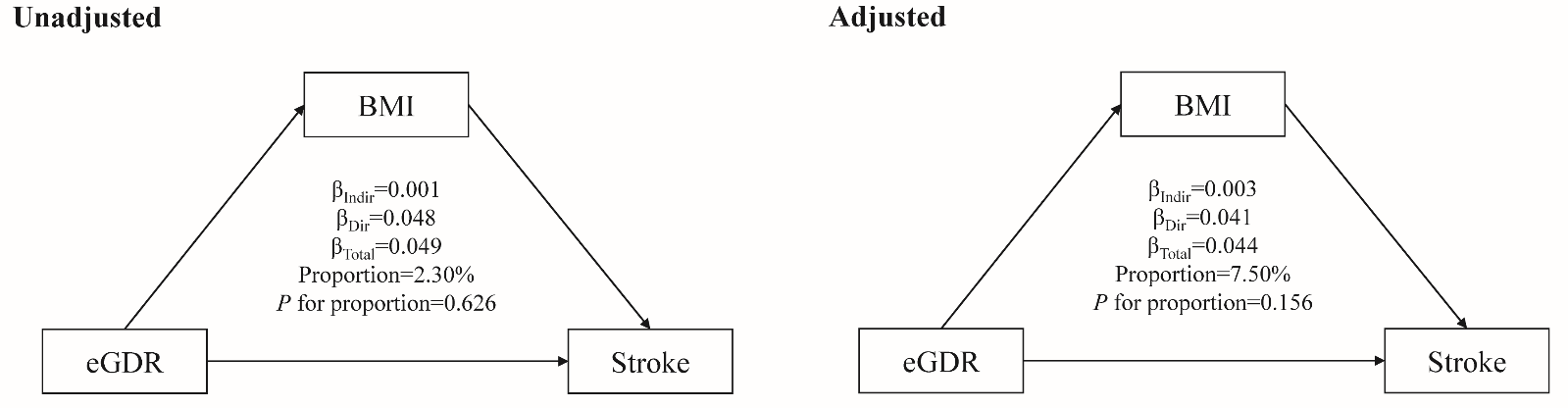


**Supplementary Figure 8** Subgroup analysis of HRs (95% CIs) for heart disease of estimated glucose disposal rate


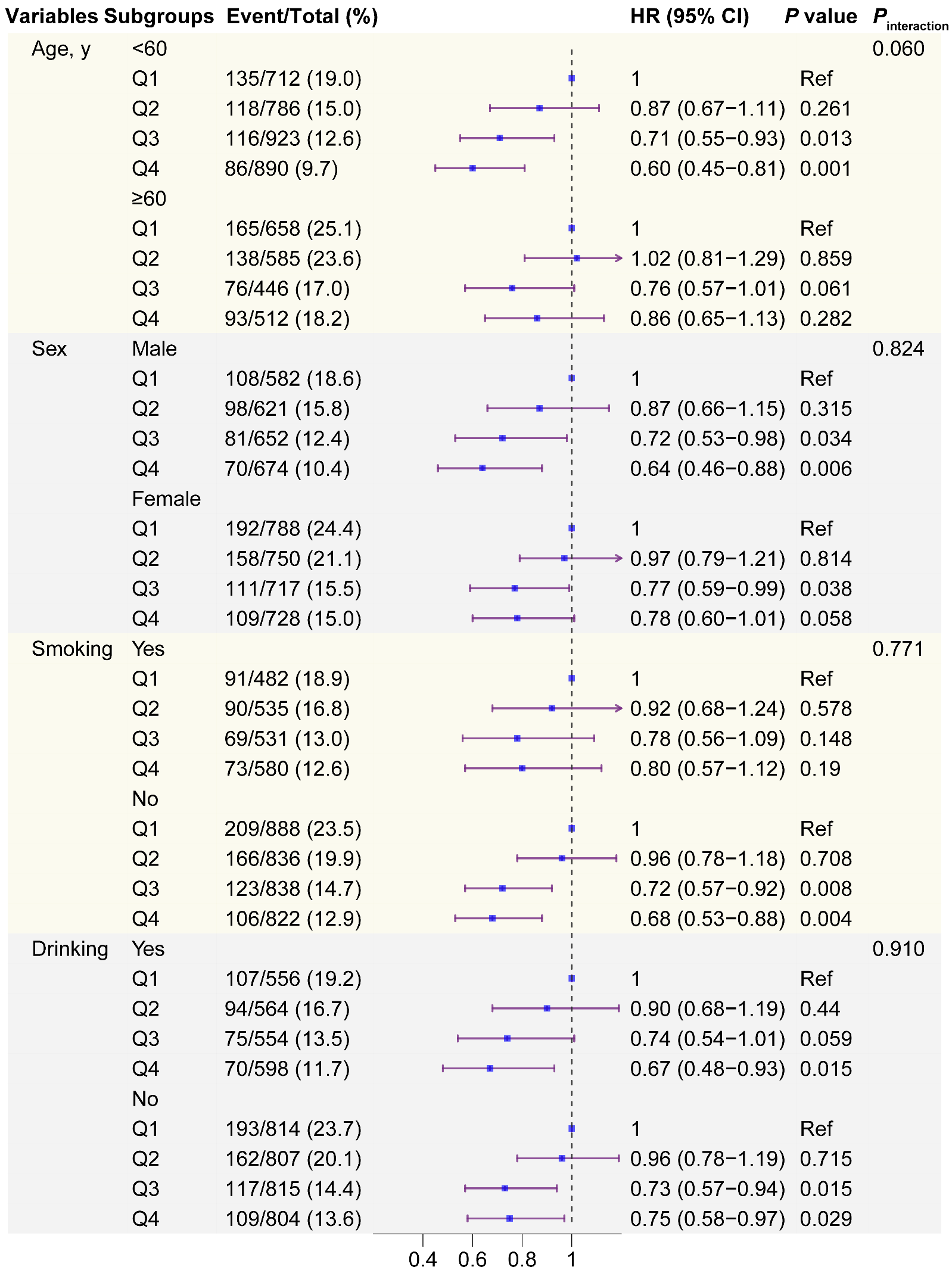


**Supplementary Figure 9** Subgroup analysis of HRs (95% CIs) for heart disease of estimated glucose disposal rate


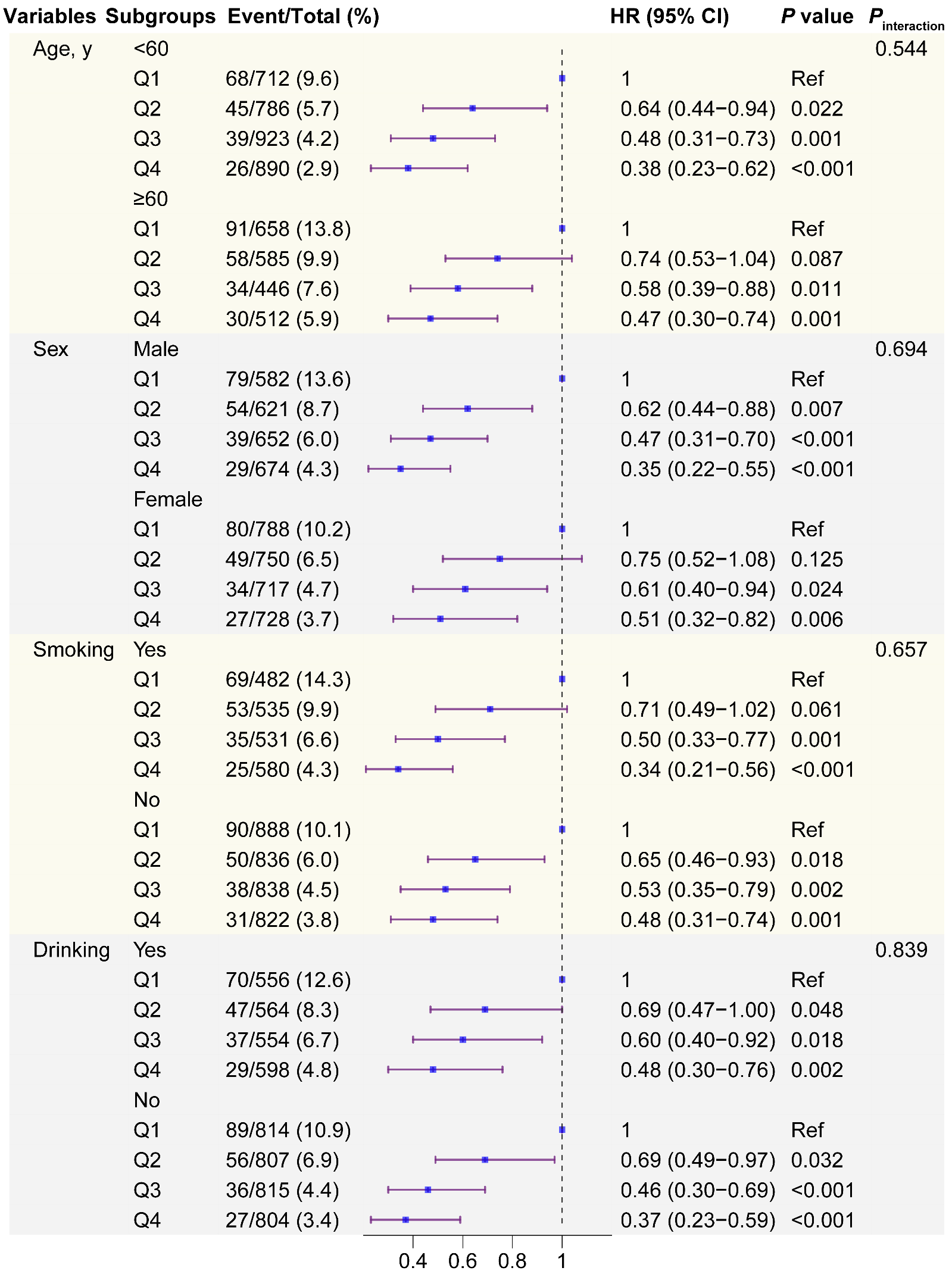


**Table S1.** Baseline characteristics of participants by outcomes

| **Characteristics** | **Overall** | **Without CVD** | **CVD** | ***P* value** |
| --- | --- | --- | --- | --- |
| n | 5512 | 4299 | 1213 |  |
| eGDR | 9.52 ± 2.05 | 9.67 ± 2.00 | 8.98 ± 2.13 | <0.001 |
| Age, years | 58.16 ± 8.82 | 57.52 ± 8.70 | 60.45 ± 8.86 | <0.001 |
| Female, n (%) | 2983 (54.1) | 2281 (53.1) | 702 (57.9) | 0.003 |
| SBP^&^, mmHg | 127.67 ± 20.60 | 126.42 ± 20.01 | 132.13 ± 22.05 | <0.001 |
| DBP^&^, mmHg | 74.65 ± 12.05 | 74.19 ± 11.82 | 76.30 ± 12.69 | <0.001 |
| BMI^&^, kg/m^2^ | 23.21 ± 3.48 | 23.08 ± 3.40 | 23.68 ± 3.72 | <0.001 |
| WC, cm | 84.47 ± 9.73 | 84.01 ± 9.45 | 86.10 ± 10.48 | <0.001 |
| Rural residence, n (%) | 3764 (68.3) | 2935 (68.3) | 829 (68.3) | 0.962 |
| Region*, n (%) |  |  |  | <0.001 |
| North | 2381 (43.2) | 1692 (39.4) | 689 (56.8) |  |
| South | 3131 (56.8) | 2607 (60.6) | 524 (43.2) |  |
| Education, n (%) |  |  |  | 0.741 |
| Junior high school and below | 5003 (90.8) | 3908 (90.9) | 1095 (90.3) |  |
| Senior high school | 470 (8.5) | 362 (8.4) | 108 (8.9) |  |
| Tertiary | 39 (0.7) | 29 (0.7) | 10 (0.8) |  |
| Marital status, n (%) |  |  |  | 0.002 |
| Married and living with spouse | 4708 (85.4) | 3706 (86.2) | 1002 (82.6) |  |
| Others | 804 (14.6) | 593 (13.8) | 211 (17.4) |  |
| Alcohol consumption, n (%) | 2272 (41.2) | 1796 (41.8) | 476 (39.2) | 0.113 |
| Smoking, n (%) | 2128 (38.6) | 1670 (38.8) | 458 (37.8) | 0.492 |
| Hemoglobin^&^, g/dL | 14.34 ± 2.20 | 14.32 ± 2.20 | 14.42 ± 2.22 | 0.190 |
| FBG, mg/dL | 99.98 ± 11.63 | 99.88 ± 11.47 | 100.30 ± 12.16 | 0.275 |
| HbA1c, % | 5.10 ± 0.40 | 5.09 ± 0.40 | 5.13 ± 0.39 | 0.012 |
| TC, mg/dL | 192.37 ± 36.96 | 191.69 ± 36.93 | 194.78 ± 36.97 | 0.010 |
| TG, mg/dl | 100.89 (72.57−144.26) | 99.12 (70.80−143.37) | 104.43 (77.00−147.79) | <0.001 |
| HDL, mg/dL | 52.50 ± 14.97 | 52.65 ± 14.97 | 51.94 ± 14.98 | 0.142 |
| LDL^&^, mg/dL | 116.88 ± 33.53 | 116.23 ± 33.32 | 119.17 ± 34.16 | 0.007 |
| BUN, mg/dL | 15.64 ± 4.38 | 15.69 ± 4.35 | 15.48 ± 4.47 | 0.128 |
| UA, mg/dL | 4.38 ± 1.20 | 4.39 ± 1.19 | 4.38 ± 1.23 | 0.807 |
| hsCRP, mg/L | 0.94 (0.52−1.96) | 0.90 (0.51−1.86) | 1.10 (0.56−2.26) | <0.001 |
| Serum creatinine, mg/dL | 0.77 ± 0.18 | 0.77 ± 0.18 | 0.77 ± 0.19 | 0.985 |
| Kidney disease, n (%) | 368 (6.7) | 263 (6.2) | 105 (8.7) | 0.002 |
| Obesity, n (%) | 556 (10.1) | 389 (9.0) | 167 (13.8) | <0.001 |

BMI, body mass index; BUN, blood urea nitrogen; CVD, cardiovascular disease; DBP, diastolic blood pressure; DM, diabetes mellitus; eGDR, estimated glucose disposal rate; FBG, fasting blood glucose; HbA1c, glycosylated hemoglobin A1c; HDL, high density lipoprotein; hsCRP, high-sensitivity C-reactive protein; LDL, low density lipoprotein; SBP, systolic blood pressure; TC, total cholesterol; TG, triglycerides; UA, uric acid; WC, waist circumference

*Region was divided into north and south based on the Qinling Mountains-Huaihe River Line

& Missing data: 43 for systolic blood pressure, 44 for diastolic blood pressure, 1 for LDL, 91 for hemoglobin, 49 for BMI.

**Table S2**. The association of estimated glucose disposal rate with cardiovascular diseases among participants with normal glucose status.

| **Variables** | **Cardiovascular disease** | | **Heart disease** | | **Stroke** | |
| --- | --- | --- | --- | --- | --- | --- |
|  | HR (95% CI) | *P* value | HR (95% CI) | *P* value | HR (95% CI) | *P* value |
| eGDR^#^ | 0.82 (0.76−0.88) | <0.001 | 0.88 (0.81−0.95) | 0.002 | 0.65 (0.57−0.74) | <0.001 |
| Q1 | Ref |  | Ref |  | Ref |  |
| Q2 | 0.90 (0.76−1.07) | 0.248 | 0.98 (0.81−1.20) | 0.873 | 0.68 (0.50−0.92) | 0.012 |
| Q3 | 0.67 (0.55−0.81) | <0.001 | 0.74 (0.60−0.93) | 0.009 | 0.49 (0.34−0.69) | <0.001 |
| Q4 | 0.61 (0.49−0.74) | <0.001 | 0.71 (0.56−0.89) | 0.003 | 0.32 (0.22−0.48) | <0.001 |

Abbreviation: BUN, blood urea nitrogen; CI, confidence interval; eGDR, estimated glucose disposal rate; HDL, high density lipoprotein; HR, hazard ratio; hsCRP, high-sensitivity C-reactive protein; LDL, low density lipoprotein; NGM, normal glucose status; Ref, reference; TC, total cholesterol; TG, triglyceride; UA, uric acid.

The model adjusted age, sex, rural residence, marital status, education, smoking, alcohol consumption status, region, TC, HDL, TG, LDL, BUN, UA, hsCRP, hemoglobin, chronic kidney disease, and obesity.

# Per SD increase

**Table S3**. The association of estimated glucose disposal rate (defined hypertension based on 130/80 mmHg) with cardiovascular diseases among participants.

| **Variables** | **Cardiovascular disease** | | **Heart disease** | | **Stroke** | |
| --- | --- | --- | --- | --- | --- | --- |
|  | HR (95% CI) | *P* value | HR (95% CI) | *P* value | HR (95% CI) | *P* value |
| eGDR^#^ | 0.82 (0.77−0.88) | <0.001 | 0.86 (0.79−0.92) | <0.001 | 0.70 (0.62−0.78) | <0.001 |
| Q1 | Ref |  | Ref |  | Ref |  |
| Q2 | 0.90 (0.77−1.06) | 0.208 | 0.98 (0.81−1.17) | 0.803 | 0.75 (0.57−0.98) | 0.033 |
| Q3 | 0.73 (0.62−0.85) | <0.001 | 0.78 (0.65−0.94) | 0.010 | 0.55 (0.41−0.73) | <0.001 |
| Q4 | 0.59 (0.49−0.71) | <0.001 | 0.67 (0.54−0.83) | <0.001 | 0.34 (0.24−0.49) | <0.001 |

Abbreviation: BUN, blood urea nitrogen; CI, confidence interval; eGDR, estimated glucose disposal rate; HDL, high density lipoprotein; HR, hazard ratio; hsCRP, high-sensitivity C-reactive protein; LDL, low density lipoprotein; NGM, normal glucose status; Ref, reference; TC, total cholesterol; TG, triglyceride; UA, uric acid.

The model adjusted age, sex, rural residence, marital status, education, smoking, alcohol consumption status, region, TC, HDL, TG, LDL, BUN, UA, hsCRP, hemoglobin, chronic kidney disease, and obesity.

# Per SD increase

**Table S4**. The association of estimated glucose disposal rate with cardiovascular diseases among participants after excluding individuals experienced CVD during or before Survey 2.

| **Variables** | **Cardiovascular disease** | | **Heart disease** | | **Stroke** | |
| --- | --- | --- | --- | --- | --- | --- |
|  | HR (95% CI) | *P* value | HR (95% CI) | *P* value | HR (95% CI) | *P* value |
| eGDR^#^ | 0.81 (0.76−0.87) | <0.001 | 0.85 (0.78−0.92) | <0.001 | 0.70 (0.63−0.79) | <0.001 |
| Q1 | Ref |  | Ref |  | Ref |  |
| Q2 | 0.88 (0.75−1.03) | 0.102 | 0.96 (0.79−1.15) | 0.637 | 0.69 (0.53−0.91) | 0.008 |
| Q3 | 0.67 (0.56−0.80) | <0.001 | 0.74 (0.60−0.91) | 0.004 | 0.54 (0.40−0.74) | <0.001 |
| Q4 | 0.61 (0.51−0.74) | <0.001 | 0.67 (0.54−0.84) | <0.001 | 0.44 (0.31−0.62) | <0.001 |

Abbreviation: BUN, blood urea nitrogen; CI, confidence interval; eGDR, estimated glucose disposal rate; HDL, high density lipoprotein; HR, hazard ratio; hsCRP, high-sensitivity C-reactive protein; LDL, low density lipoprotein; NGM, normal glucose status; Ref, reference; TC, total cholesterol; TG, triglyceride; UA, uric acid.

The model adjusted age, sex, rural residence, marital status, education, smoking, alcohol consumption status, region, TC, HDL, TG, LDL, BUN, UA, hsCRP, hemoglobin, chronic kidney disease, and obesity.

# Per SD increase

**Table S5**. The association of estimated glucose disposal rate with cardiovascular diseases among non-DM participants (defined DM based on FBG and HbA1c).

| **Variables** | **Cardiovascular disease** | | **Heart disease** | | **Stroke** | |
| --- | --- | --- | --- | --- | --- | --- |
|  | HR (95% CI) | *P* value | HR (95% CI) | *P* value | HR (95% CI) | *P* value |
| eGDR^#^ | 0.83 (0.78−0.88) | <0.001 | 0.87 (0.81−0.93) | <0.001 | 0.69 (0.62−0.76) | <0.001 |
| Q1 | Ref |  | Ref |  | Ref |  |
| Q2 | 0.84 (0.72−0.97) | 0.018 | 0.89 (0.75−1.05) | 0.174 | 0.69 (0.54−0.88) | 0.003 |
| Q3 | 0.69 (0.58−0.81) | <0.001 | 0.76 (0.63−0.91) | 0.004 | 0.51 (0.38−0.68) | <0.001 |
| Q4 | 0.65 (0.55−0.78) | <0.001 | 0.73 (0.60−0.89) | 0.002 | 0.40 (0.29−0.55) | <0.001 |

Abbreviation: BUN, blood urea nitrogen; CI, confidence interval; eGDR, estimated glucose disposal rate; FBG, fasting blood glucose; HbA1c, glycosylated hemoglobin A1c; HDL, high density lipoprotein; HR, hazard ratio; hsCRP, high-sensitivity C-reactive protein; LDL, low density lipoprotein; NGM, normal glucose status; Ref, reference; TC, total cholesterol; TG, triglyceride; UA, uric acid.

The model adjusted age, sex, rural residence, marital status, education, smoking, alcohol consumption status, region, TC, HDL, TG, LDL, BUN, UA, hsCRP, hemoglobin, chronic kidney disease, and obesity.

# Per SD increase
